# Operations research and analytics to combat human trafficking: A systematic review of academic literature

**DOI:** 10.1371/journal.pone.0273708

**Published:** 2022-08-29

**Authors:** Geri L. Dimas, Renata A. Konrad, Kayse Lee Maass, Andrew C. Trapp

**Affiliations:** 1 Data Science Program, Worcester Polytechnic Institute, Worcester, MA, United States of America; 2 Business School, Worcester Polytechnic Institute, Worcester, MA, United States of America; 3 Mechanical and Industrial Engineering Department, Northeastern University, Boston, MA, United States of America; University of Hong Kong, HONG KONG

## Abstract

Human trafficking is a widespread and compound social, economic, and human rights issue occurring in every region of the world. While there have been an increasing number of anti-human trafficking studies from the Operations Research and Analytics domains in recent years, no systematic review of this literature currently exists. We fill this gap by providing a systematic literature review that identifies and classifies the body of Operations Research and Analytics research related to the anti-human trafficking domain, thereby illustrating the collective impact of the field to date. We classify 142 studies to identify current trends in methodologies, theoretical approaches, data sources, trafficking contexts, target regions, victim-survivor demographics, and focus within the well-established 4Ps principles. Using these findings, we discuss the extent to which the current literature aligns with the global demographics of human trafficking and identify existing research gaps to propose an agenda for Operations Research and Analytics researchers.

## Introduction

Human trafficking (HT) involves the commercial exchange and exploitation of individuals for monetary or other gain using force, fraud, or coercion [1] and is a widespread social, economic, and human rights issue. While the trafficking of individuals is a centuries-old phenomenon, over the past two decades there has been growing public and research awareness, in part with the ratification of the 2000 Palermo Protocol to Prevent, Suppress, and Punish Trafficking in Persons [2]. Although precise figures are elusive, the Global Estimates of Modern Slavery Report estimates that HT impacts 25M individuals and annually generates more than 150 billion USD in illicit gains globally [3, 4]. HT is broadly classified as labor and sex trafficking; while all trafficking features exploitation, the actions and means by which HT occurs may differ [5]. Labor trafficking takes place in a wide variety of sectors, including the agriculture, domestic work, construction, fishing, food service, and beauty industries. Sex trafficking is a part of the broader commercial sex industry, occurring in industries such as escort services, brothels, and pornography.

Because the scope of HT activity is vast and there are diverse ways in which individuals are exploited [6], context is critical, and effectively addressing HT increasingly requires efforts from multiple disciplines, including interdisciplinary collaborations. For example, HT interventions include approaches from multiple sectors and disciplines such as social work [7–9], healthcare [10, 11], criminal justice [12–14], and economics [15, 16]; each domain brings unique perspectives and methods to understand and address HT.

Owing to the breadth of domains that contribute to anti-HT research, a wealth of literature exists that has been well-documented in surveys over the years [10, 11, 17–21]. Existing reviews focus on social science, healthcare, and law enforcement approaches; whereas OR and Analytics have much to offer [22], no systematic review exists for the emerging landscapes of Operations Research (OR) and Analytics as applied to anti-HT.

The present study identifies and classifies the existing OR and Analytics literature related to anti-HT operations. Building off the earlier work of Krammer-Kerwick et al. [23] and Caulkins et al. [24], this systematic review proposes an agenda for future research in this field, filling a gap in the current literature. This study focuses on the four broad principles of anti-HT: prevention, protection, prosecution, and partnership (4Ps) [25], extending their definition in relation to the OR and Analytics fields. We examine the following research questions:

(i) What aspects of HT are being studied by OR and Analytics researchers?
(ii) What OR and Analytics methods are being applied in the anti-HT domain?
(iii) What are the existing research gaps associated with (i) and (ii)?

We organize the remainder of our study as follows. In the method of collection and categorization section we define the scope of this review, and in the data section we define the data features for analysis. In the implications and observations section we discuss the implications of the survey and, based on the observed gaps, suggest areas for future work. We conclude our study in the final section.

## Method of collection and categorization

**Fig 1:**
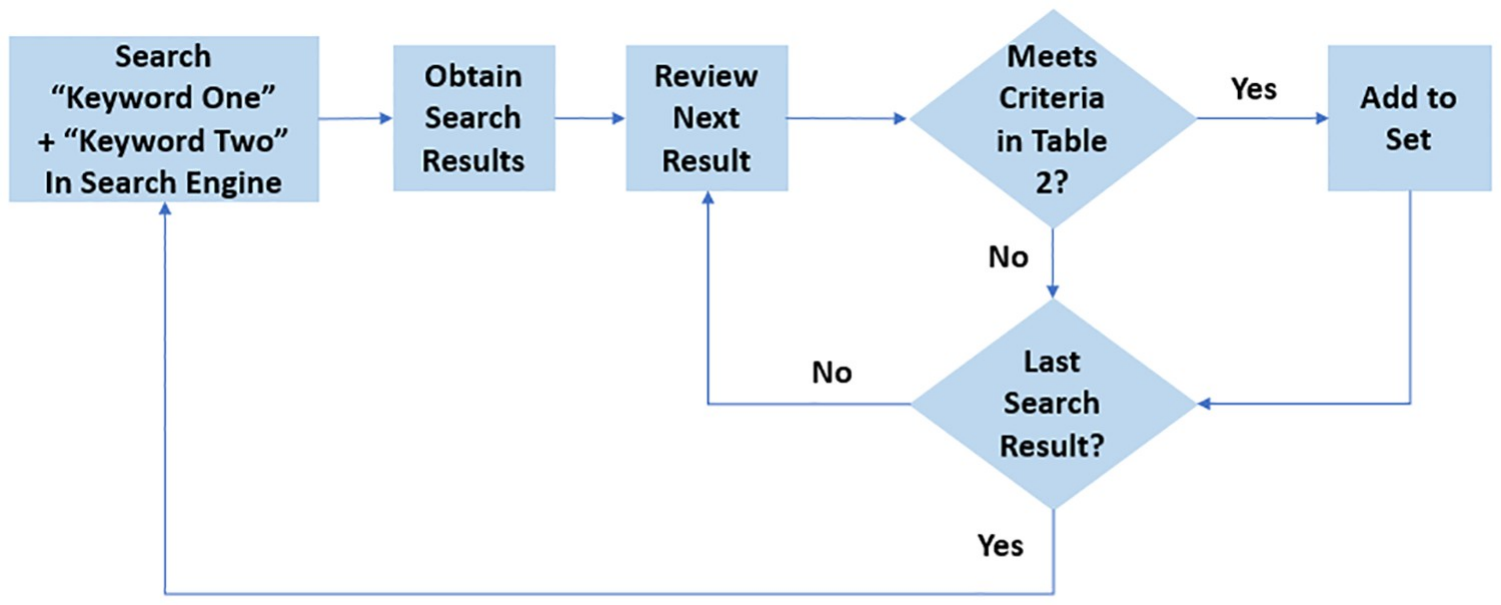
Heuristic process of data collection.

We conducted a systematic literature review inventorying studies to answer the three research questions outlined in the introduction. The methodology used for this systematic review was guided by the Preferred Reporting Items for Systematic Reviews and Meta-Analyses (PRISMA) [26]. The collection process (Fig 1) was based on keyword searches that generated a set of research studies for analysis. Two sets of search words were defined using the combined knowledge of the authors on HT terminology and OR and Analytics methods. These keywords were used in a procedure to identify and select studies that met a set of pre-defined criteria. The first set of keywords reflects terms related to HT, while the second reflects common methods in the OR and Analytics fields (see Table 1). The search and selection of studies was performed by the lead author (G.L.D.), and any uncertainty regarding a study’s inclusion was resolved through discussion with the coauthors.

**Table 1:** Keywords used in search process.

Keyword One: Human Trafficking Related	
Child Labor / Child Labour	Debt Bondage
Domestic Servitude	Forced Labor/ Forced Labour
Human Trafficking	Labor Trafficking / Labour Trafficking
Modern Slavery	Sex Trafficking
Trafficking in Persons	
Keyword Two: Methodology Related	
Clustering/Classification	Data Envelopment Analysis
Data Science	Game Theory
Graph Theory/Construction	Information Extraction
Integer Programming	Machine Learning
Natural Language Processing	Network Interdiction/Flow
Operations Research	Queueing/Queueing Theory
Resource Allocation	Simulation
Simulation	Supervised/Unsupervised Learning
Supply Chain	Web Crawling

**Fig 2:**
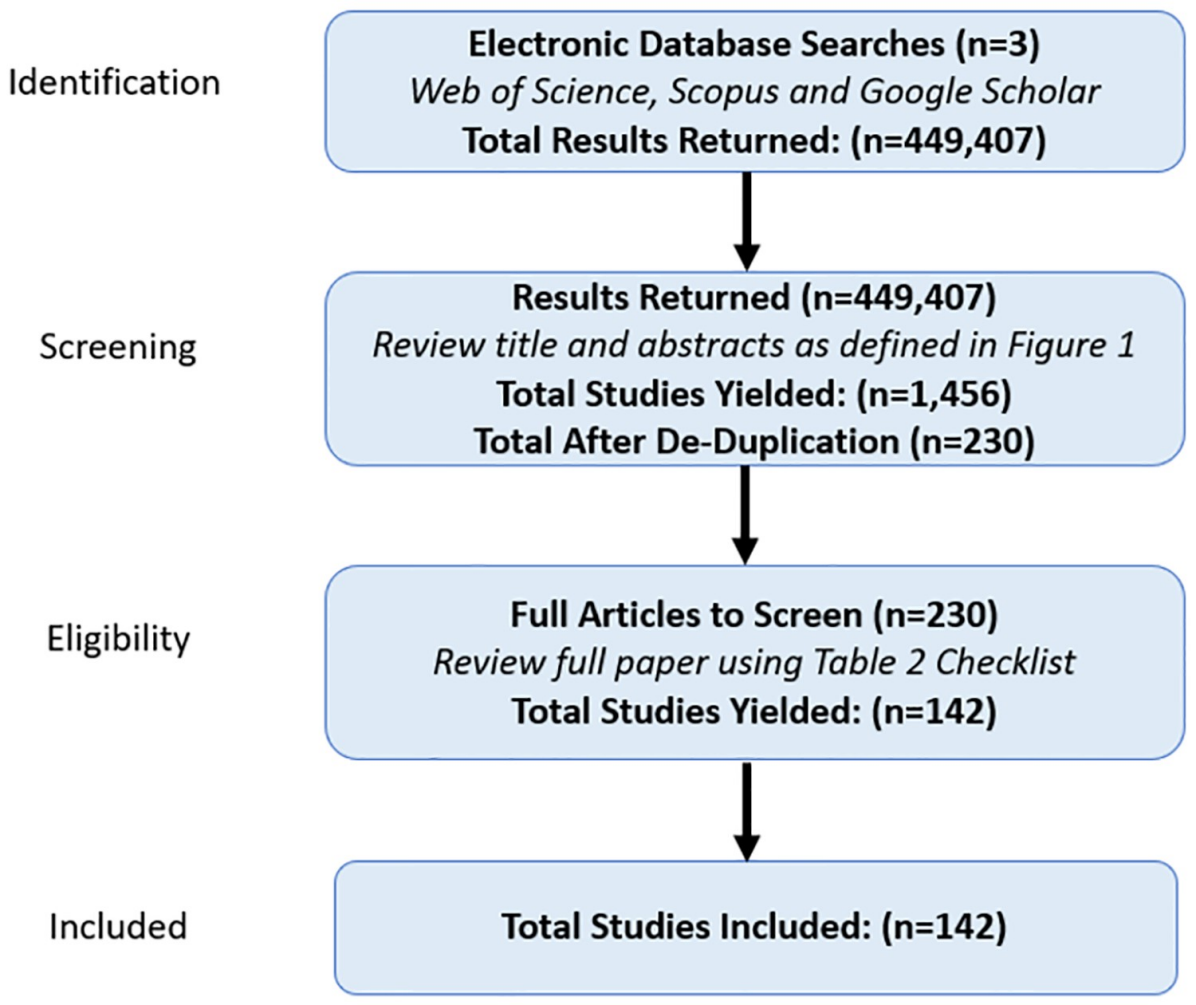
Overview of selection process.

Each search query followed the format: “Keyword One” + “Keyword Two” (such as “Debt Bondage” AND “Integer Programming”), each keyword pair was applied across three bibliographic databases: Scopus, Web of Science, and Google Scholar. The database search was conducted from June 2021 through March 2022. The search results were truncated to studies available through the end of 2021 to provide a comparable year-over-year basis for the research landscape. The sum of these two searches resulted in a total of 449,407 studies for potential inclusion. After the keyword search identification process, a two-step selection process was followed (see Fig 2). An initial screening process was conducted that evaluated the search results returned for each query, where titles and abstracts were screened and added to the set based on the criteria outlined in Table 2. The stopping criteria for each keyword pair search followed a heuristic approach: if at least 50 results returned no eligible results, then the current keyword search was stopped and the next keyword search began. The intuition behind this approach is that by design, search engines return the most relevant results first, and therefore if after a certain point no relevant results are produced (at least 50 results in our context), then it is highly unlikely relevant results exist past that point. After the initial screening process, the set included 230 unique studies for review. A more in-depth review using the eligibility requirements checklist (Table 2) was followed in step two for each of the 230 studies.

**Table 2:** Eligibility requirements checklist.

Requirements Checklist
Main contribution or focus fell into one of the following three themes: *Methodological* Operations Research orientation *Methodological* Analytics orientation (Data Science, or other Applied Analytics) *Position / Thought* pieces in Operations Research or Analytics Main application or case study was on anti-HT efforts Only studies, articles, theses and dissertations were kept (e.g., no books, workshops or government reports) If multiple versions of a study exist (such as a conference paper followed by a peer-reviewed journal article) only the most recent, comprehensive version was kept If the study appeared as a section in one study but was further developed into a full study, only the full study was kept

First, only studies that fell into one of the three themes: *Operations Research* methodologies, *Analytical* methodologies, or *Position / Thought* pieces related to Operations Research and Analytics were included. Second, only studies whose primary application area was anti-HT and was written completely in English text were included. Third, book chapters, workshops, and reports (governmental, intragovernmental, non-government organizations, think-tanks) were excluded. Finally, if multiple studies related to a single work were found, only the most complete version were included. Peer-reviewed studies, dissertations, and pre-print studies were included to produce a full and comprehensive review of the current research landscape. The full article screening process resulted in a total of 142 studies included in the set for final review.

As with any search process based upon a predefined set of keywords and checklist requirements, the 142 identified studies may not be exhaustive in scope. However, given our collective experience in researching at the intersection of anti-HT and OR and Analytics, we believe the generated set of studies is representative of current literature at this confluence. A repository containing our classification data can be found publicly at https://github.com/gldimas/Dimas-et.-al-2022_Human-Trafficking-Literature-Review.

While the focus of this study was to capture the general trends in anti-HT research within the OR and Applied Analytics communities, we acknowledge there exist many relevant studies that either fall outside of the scope of the present analysis (such as reports) or focus on the problem domain of anti-HT but do not have a specific OR or Analytics-related attribution, and therefore were excluded. We have included such studies in the S1 File and further point the reader to reviews such as Raets and Janssens [27], Farrell and De Vries [28], and Weitzer [29] to better capture the broader scope of anti-HT research.

## Data

The classification of studies was independently conducted by the lead author (G.L.D.) who throughout the process conferred with all coauthors. Each of the 142 studies in the set were reviewed and assigned labels to nine key features: **Publication Year**, **Category**, **Context**, **Demographics**, **Target Region**, **Data Source**, **Theoretical Approach**, **Methodologies**, and **4Ps**. We next explain each feature and its respective values.

**Publication Year**: Observed years were **2010—2021**, inclusive. Only studies available through December 2021 were included in our scope to allow for comparison across complete calendar years. This feature offers valuable information about the progress and patterns of research in OR and Analytics over more than ten years.

**Category**: We considered three categories: **Operations Research (OR), Analytics**, and **Position / Thought**. Whereas the first two categories are distinguished by their *methodological* focus, the latter includes *position / thought* pieces from either the OR or Analytics domains. We required a single category to be assigned to each study, and thus selected the category that we felt best matched the primary theme of the work.

**Context**: We classified the primary topical HT area (as stated or inferred) into three contexts: **Sex, Labor**, and **General**. If the application was not specified, we assumed it to be general and thus applicable to both sex and labor trafficking.

**Demographics**: We classified studies into five demographic groups based on the population of interest (such as victims, potential victims, and survivors): **Female, Male, Child, LGBTQ+**, and **Unspecified / All Individuals**. If no specific demographic characteristics were stated or could be inferred, we assumed it to be applicable to all individuals. A study could be classified into multiple demographics such as a study focused on female children.

**Target Region**: We subdivided the geographic location specified either by the data used in the study or by the region discussed in the background of the study, into world regions: **Africa, Asia, Australia/Oceania, Europe, North America, South America, Unspecified / All regions**. A study could cover multiple geographic locations and therefore have multiple target regions identified.

**Data Source**: We classified the type of data used in the study into four categories: **Primary, Secondary, Mixed, N/A**. Primary data are collected directly from anti-HT organizations or researchers, including interviews and surveys. Secondary data are data that have already been collected for other purposes or are publicly available such as data from websites hosting illicit advertisements (such as backpage.com and rubmaps.com) and government reports. While many studies used their own methodologies to scrape public data sources such as escort and massage websites, we still consider these to be secondary sources. We classify studies utilizing expert judgements for determining data estimates to be secondary data. Mixed data means the study used both primary and secondary data in their work, and N/A indicates data was not used in the study.

**Theoretical Approach**: We classified the central theoretical approach to address HT into six categories: **Decision Support, Inferential Statistics / Detection, Network Flow, Resource Allocation, Supply Chain**, and **Other / Unspecified**. Decision Support explores ways to inform decision makers about initiatives to improve and better address HT, often building tools or systems for practitioners to use. Inferential Statistics / Detection focuses on identifying, estimating, or inferring aspects of HT. Network Flow studies are related to the flow of individuals and possibly trafficking network interaction. Resource Allocation addresses the use and allocation of resources in anti-HT efforts. Supply Chain studies examine the supply and demand of HT within a network. If a study approaches HT from a theoretical approach not listed, we label these studies as Other / Unspecified. A study may be classified under multiple theoretical approaches.

**Methodologies**: We classified the main methodologies used in the set of studies into 21 categories:

- Active Learning
- Clustering or Classification
- Data Envelopment Analysis
- Empirical Analysis
- Facility Location
- Game Theory
- Graph Construction
- Investigative Search
- Link Inference
- Machine / Deep Learning (General)
- Natural Language Processing
- Information Extraction
- Integer Programming
- Network / Graph Theory
- Network Interdiction
- Queueing Theory
- (Social) Network Analysis
- Simulation
- Unsupervised or Minimally Supervised Learning
- Web Crawling / Scraping
- Other

We ascribe methods to a study based on the introduction, conclusion, and main method or focus throughout the study. A study may apply a variety of different methods and therefore be classified into multiple methodologies.

**4Ps**: Activities to fight HT are often discussed under four broad principles: prevention, protection, prosecution, and partnership. These principles are collectively referred to as the 4Ps—a well-recognized classification within the anti-HT community [22]. A study may be classified under multiple principles. The 4Ps naturally correspond with efforts in the social science, healthcare, and law enforcement disciplines, and their alignment with OR and Analytics works is less evident. Thus, we adapt the 4Ps definitions to define each as it relates to OR and Analytics using the collective knowledge and experience of the authors in the anti-HT and OR and Analytics fields. To the best of our knowledge, this is the first attempt to define each of the 4Ps as it relates specifically to the OR and Analytics fields and constitutes an important contribution of this study. Prevention, Protection, and Prosecution were originally referred to as the 3P paradigm [25] which has since been informally expanded to include a fourth “P” representing Partnership. Prevention refers to efforts focused on a proactive approach to prevent trafficking such as awareness campaigns and education; Prosecution refers to efforts to punish traffickers; and Protection involves meeting post-trafficking victim needs such as counseling, job training, housing, and other support to facilitate survivor recovery and restoration.

Partnership was introduced to serve as a complementary means to further improve the efficacy among the 3Ps, enlisting all segments of society in the fight against HT [25]. Together the 4Ps capture the spectrum of efforts in combating HT and therefore are an important feature for our literature review. Accordingly, we have adapted these 4Ps and classified studies in the following manner:

- **Prevention**: The goal of the study is the prevention of HT either now or in the future and assumes no trafficking is currently taking place. Such studies typically feature victim-centric methodologies to help potential victims avoid being trafficked, such as awareness campaigns and education. Studies that consider reducing the re-trafficking risk of survivors who have left their trafficking environment also fall within the scope of the prevention principle.
- **Protection**: The goal of the study is to protect and aid the survivor during and post-exploitation. We consider victim-driven detection and disruption of HT networks to be a form of protection, as the focus of the study is mitigating the risk to an individual of further exploitation (including studies that consider NGOs, healthcare, and other non-law enforcement detection).
- **Prosecution**: The goal of the study is to aid the prosecution of traffickers (often from a law enforcement perspective). We consider detection and disruption of HT networks aimed at locating, understanding, and stopping traffickers under the prosecution principle.
- **Partnership**: The goal of the study is to show the benefit of collaboration and data sharing across different sectors, countries, or groups working together toward the common goal of addressing one or more areas of HT.

## Implications and observations

**Fig 3:**
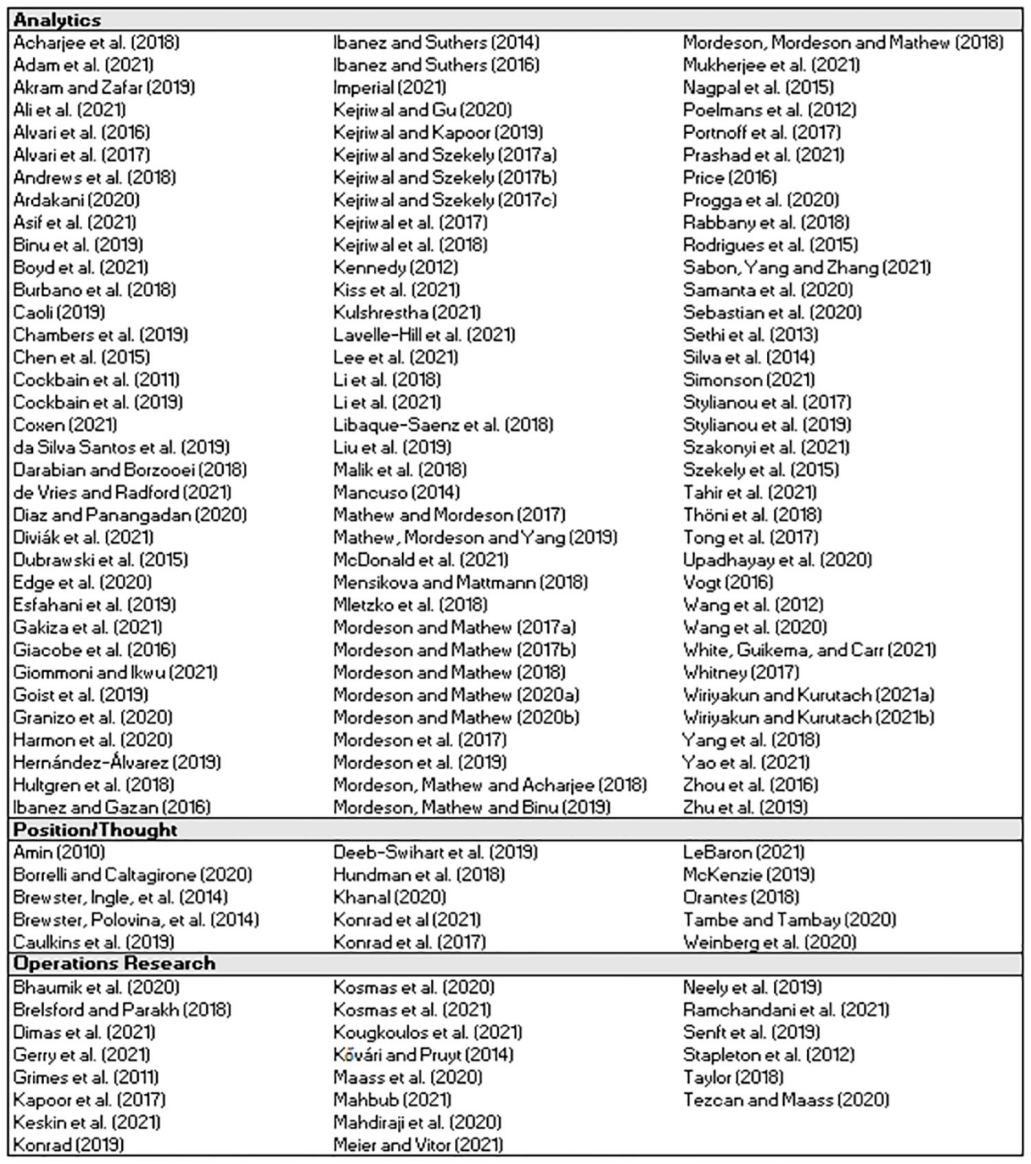
Category classification for the set of 142 studies.

**Fig 4:**
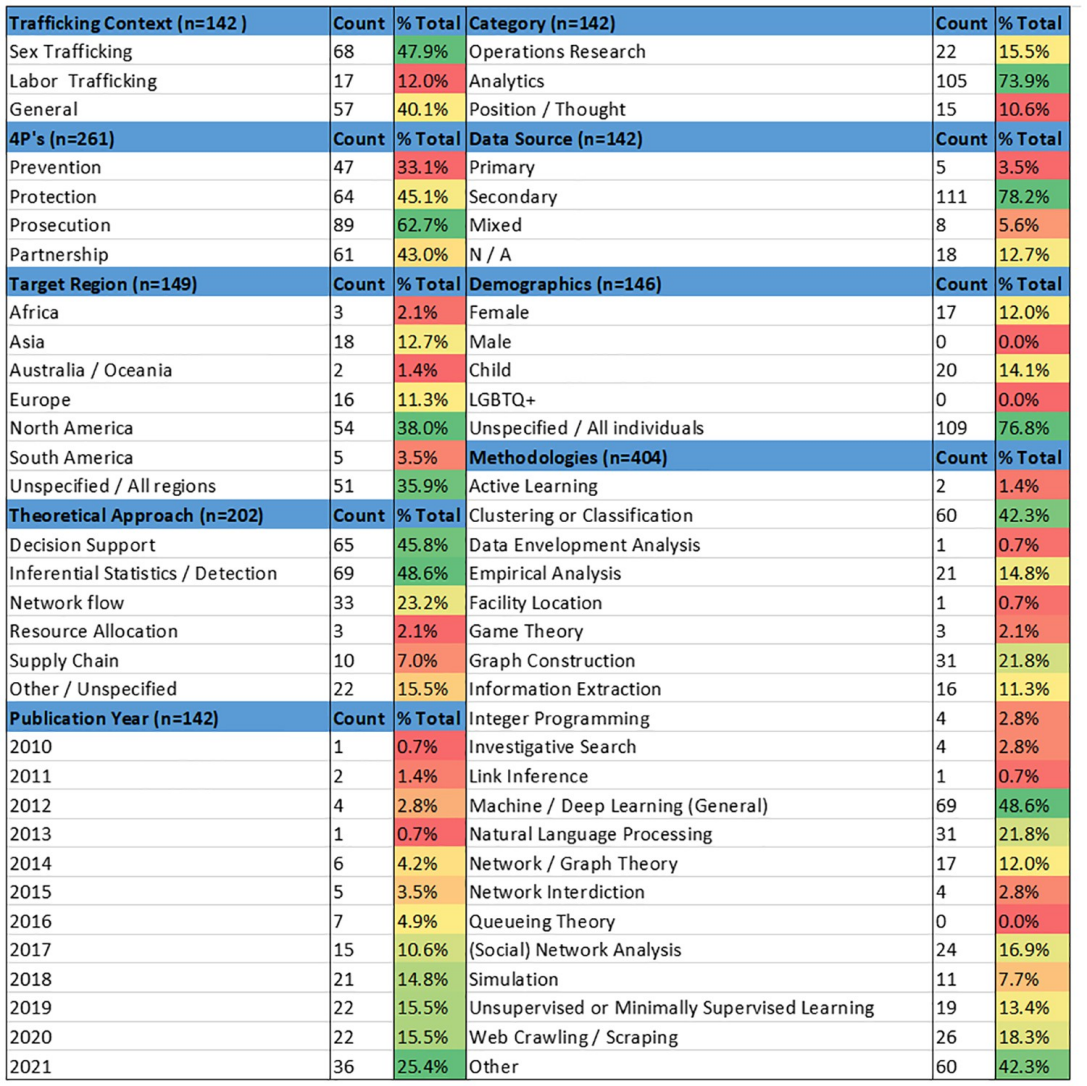
Summary statistics for the set of 142 studies.

Of the 142 studies in the set, the majority (73.9%) were categorized as Analytics [30–134], with 15.5% classified as Operations Research [135–156] and 10.6% as Position / Thought [22, 24, 157–169]. Fig 3 depicts this breakdown. Fig 4 provides summary statistics on each of the nine key features. All percentages are calculated in relation to the total number of studies in the set (142) unless stated otherwise. As some studies contained multiple methods or were identified to have multiple values within a feature, a feature may not always sum to 100.0%.

### Research question 1: What aspects of HT are being studied?

**Fig 5:**
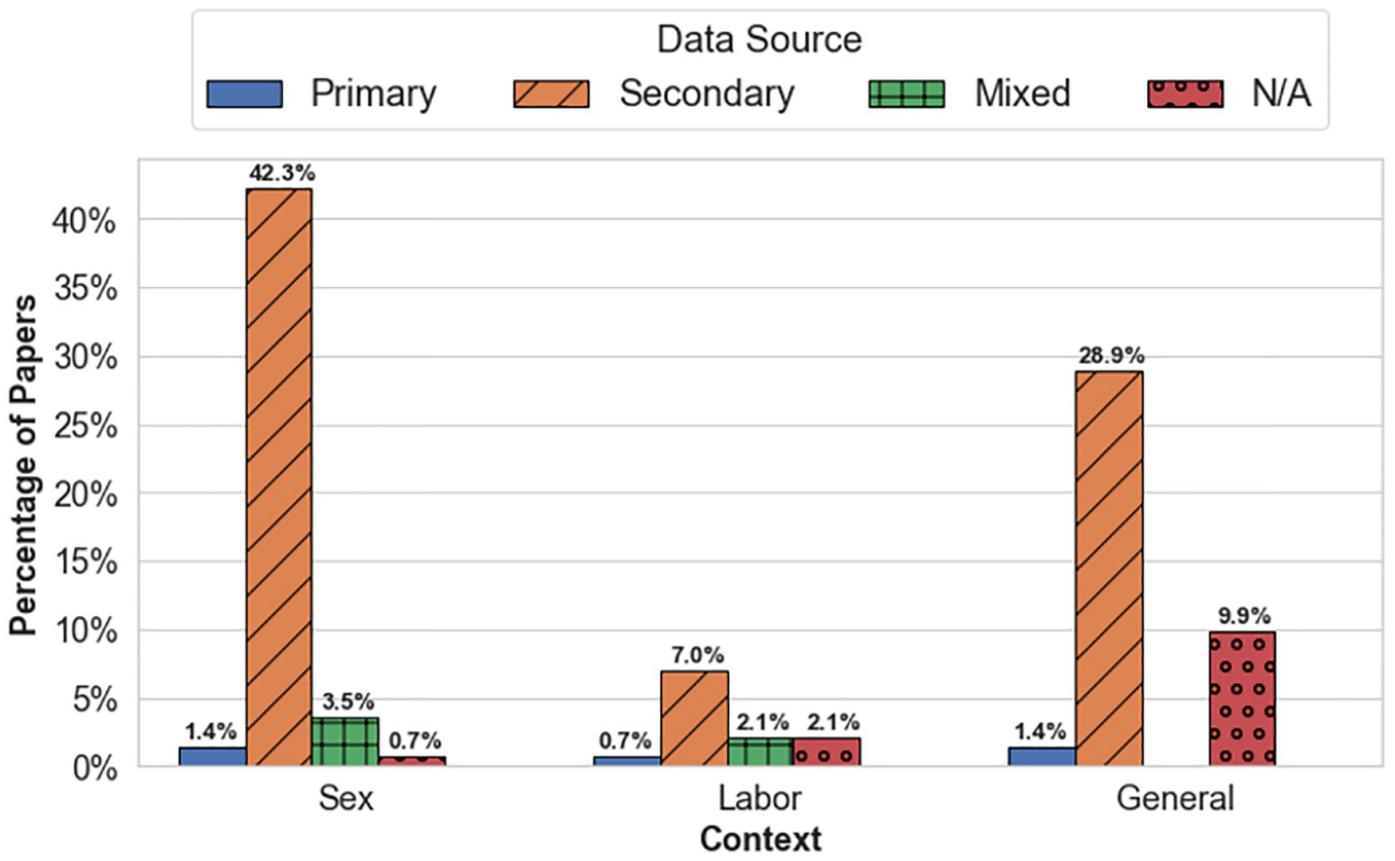
Context of HT and data source.

**Fig 6:**
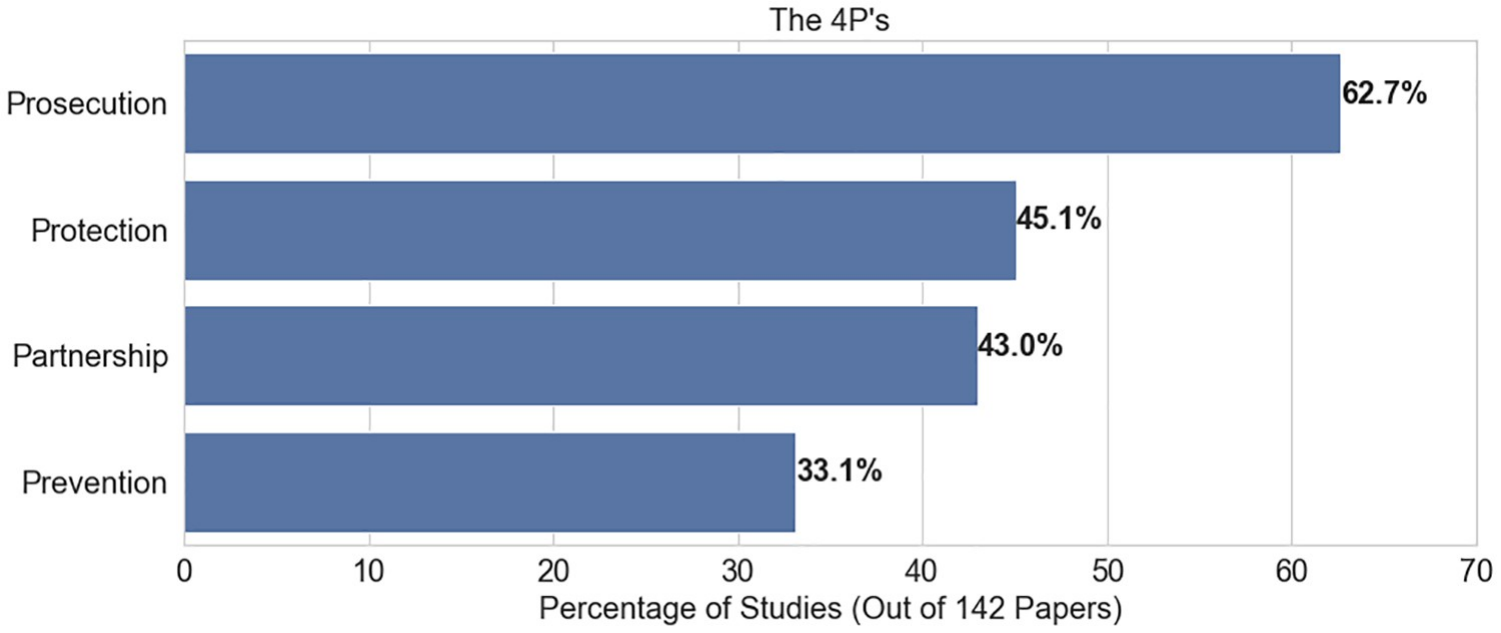
Percent of studies involving 4Ps.

Although both sex and labor trafficking have been addressed in the OR and Analytics literature, an overwhelming number of studies focus specifically on sex trafficking. Fig 4 illustrates the inclination of OR and Analytics studies to focus on sex trafficking (4 7.9%), with only 1 2.0% concentrating on labor trafficking, while 4 0.1% apply to general (both) trafficking contexts. As observed in Fig 5, studies overwhelming use secondary data, with fewer than 4.0% using a primary data source. The use of secondary data is likely due to accessibility; almost all studies on sex trafficking (60.3%) used data pulled from escort websites (or other online sites hosting illicit advertisements) which are public and therefore easier to access. The use of escort websites (in particular, backpage.com) as a source of data result in over 3 8.0% of the studies focusing on the North American region. Although the United States Department of Justice shut down backpage.com in 2018 [170], other escort and massaging sites offer illicit services and constitute the data source for several studies. Remarkably, 7 6.8% of studies were not tailored to a specific demographic, despite the differences between typologies and demographics of victims [6]. From the 4Ps perspective, prosecution is the most common principle (62.7%) among all studies, with considerably less focus on partnership, protection, and prevention (Fig 6). A single study may be categorized under multiple 4Ps principles, and therefore the values in Fig 6 do not sum to 100.0%.

### Research question 2: What OR and analytical methods are being applied in the anti-HT domain?

Fig 4 provides summary statistics such as counts and percentages for the frequency each value was observed in the set of studies. While the percentages are calculated based on the 142 studies in the set, because a study may belong to multiple values within a feature, the total observations within each feature are provided in the parenthesis. The colors highlight the magnitude of studies within each feature value, with green indicating higher percentages, and red lower percentages. A study may fall under more than one value and therefore the percentages will not always sum to 100.0%. Methods related to machine learning (Machine / Deep Learning (General), Clustering or Classification, Unsupervised / Minimally Supervised Learning, Natural Language Processing, and Active Learning) were observed in over half of the studies. Machine / Deep Learning (General) and Clustering or Classification were the two most popular methods, accounting for about 32.0% of all methods observed (out of the total 404 methods identified, see Fig 4). Web Crawling / Scraping was used to generate a secondary dataset for analysis in 18.3% of the studies, reflecting our previous observations in Research question 1 regarding the high use of secondary data extracted from massage and escort websites. At least one method for the construction and analysis of networks (Graph Construction, Network / Graph Theory, Network Interdiction and (Social) Network Analysis) were observed in 34.5% of the studies, with most emphasizing Graph Construction.

The observed theoretical approaches are closely related to specific methods. For example, nearly half of the studies focused on Inferential Statistics / Detection or Decision Support, most of which applied various machine learning methods. Network Flow methods appear in nearly 24.0% of studies, specifically Graph Construction and (Social) Network Analysis.

**Fig 7:**
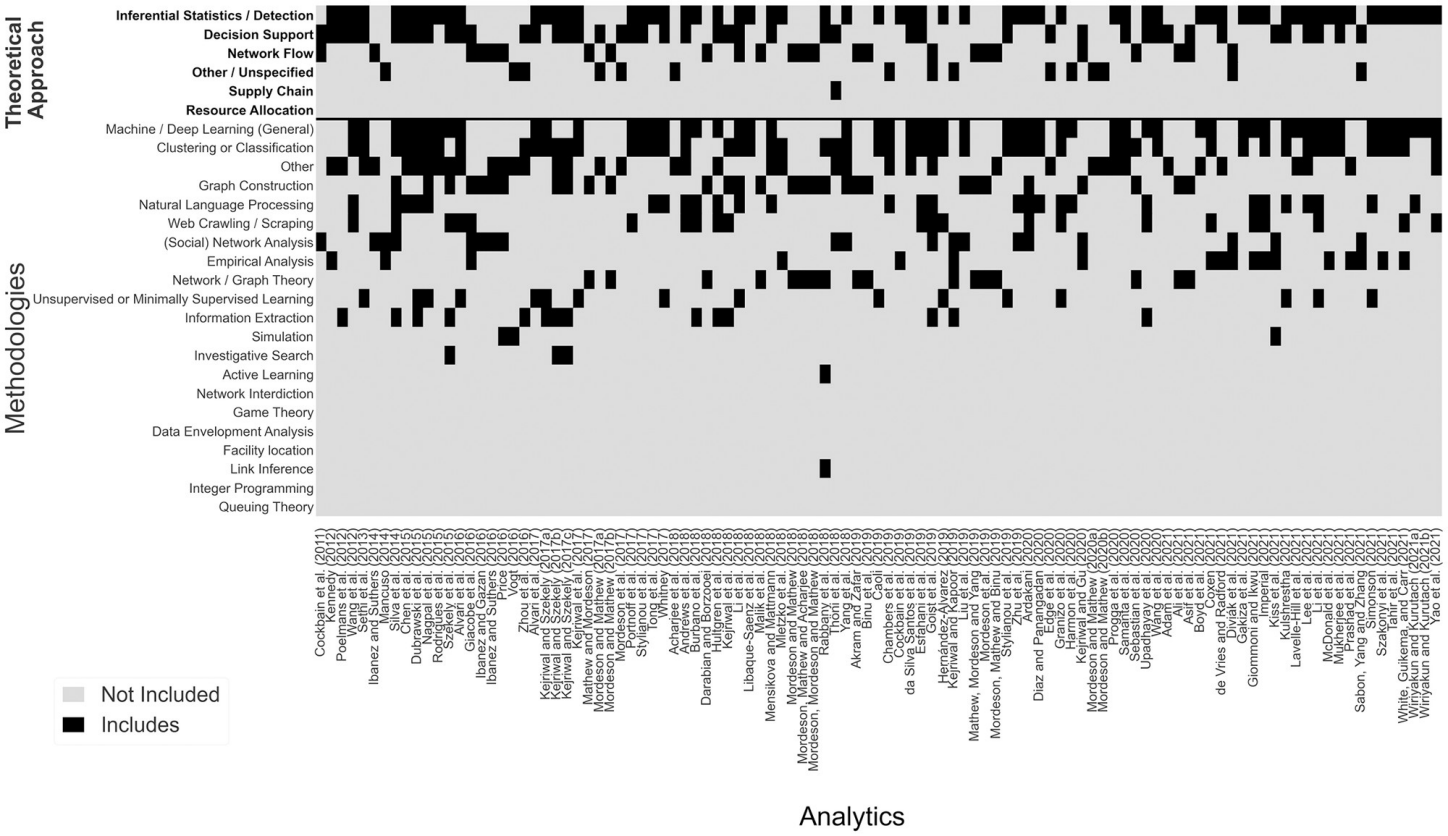
Granular view of theoretical and methodological topic inclusion for the set of studies categorized as analytics. The *x*-axis lists each study; and the *y*-axis depicts each of the Theoretical Approaches and Method. If a study includes a given feature value, the box is black, and grey otherwise. Theoretical approaches and methods are sorted in descending order based on the total count for each row.

**Fig 8:**
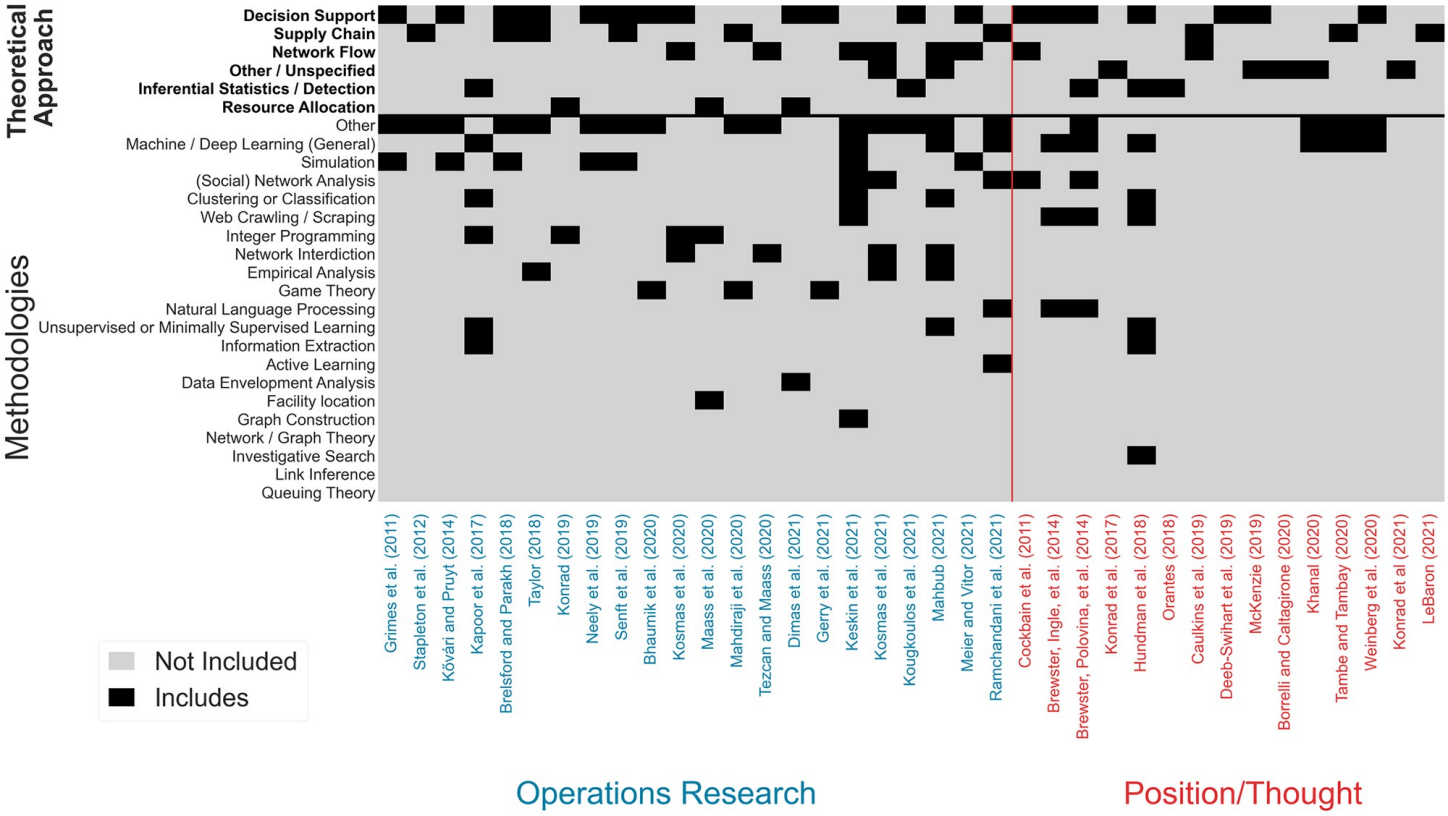
Granular view of theoretical and methodological topic inclusion for the set of studies categorized as operations research or position / thought. The *x*-axis lists each study; and the *y*-axis depicts each of the Theoretical Approaches and Method. Operations Research studies appear on the left (in blue), and Position / Thought studies appear on the right (in red). If a study includes a given feature value, the box is black, and grey otherwise. Theoretical approaches and methods are sorted in descending order based on the total count for each row.

Figs 7 and 8 depict each study on the *x*-axis. In Fig 7 we display all studies categorized as Analytics, and in Fig 8 we categorize studies on the left as Operations Research (in blue), and studies on the right as Position / Thought studies (in red). For each study, the top six *y*-axis labels indicate its Theoretical Approach(es), while the remaining 21 following labels indicate methods used. If a study includes a given feature value, the box is black, and grey otherwise. Theoretical approaches and methods are sorted in descending order based on the total count for each row. Fig 7 indicates that the majority of studies (over 77.0%) in the Analytics category take an Inferential Statistics / Detection or Decision Support theoretical approach. Fig 8 shows that studies in the Operations Research category are diverse, addressing the problem from distinct theoretical approaches and applying a variety of methodologies. Studies categorized as Position / Thought address a variety of theoretical approaches and topics, which is a good indication that OR and Analytics researchers are exploring HT from different fields.

While Resource Allocation and Supply Chain roughly make up only 9.0% of all studies, they account for over 40.0% within the Operations Research category. Looking more closely at the relationship between Web Crawling / Scraping and Clustering and Classification methods we see a large majority (nearly 70.0%) of Web Crawling / Scraping studies apply Clustering and Classification methods. In addition, more than 72.0% of studies that applied both Web Crawling / Scraping and Clustering and Classification shared the goal of identifying sex trafficking in online advertisements or tweets.

**Fig 9:**
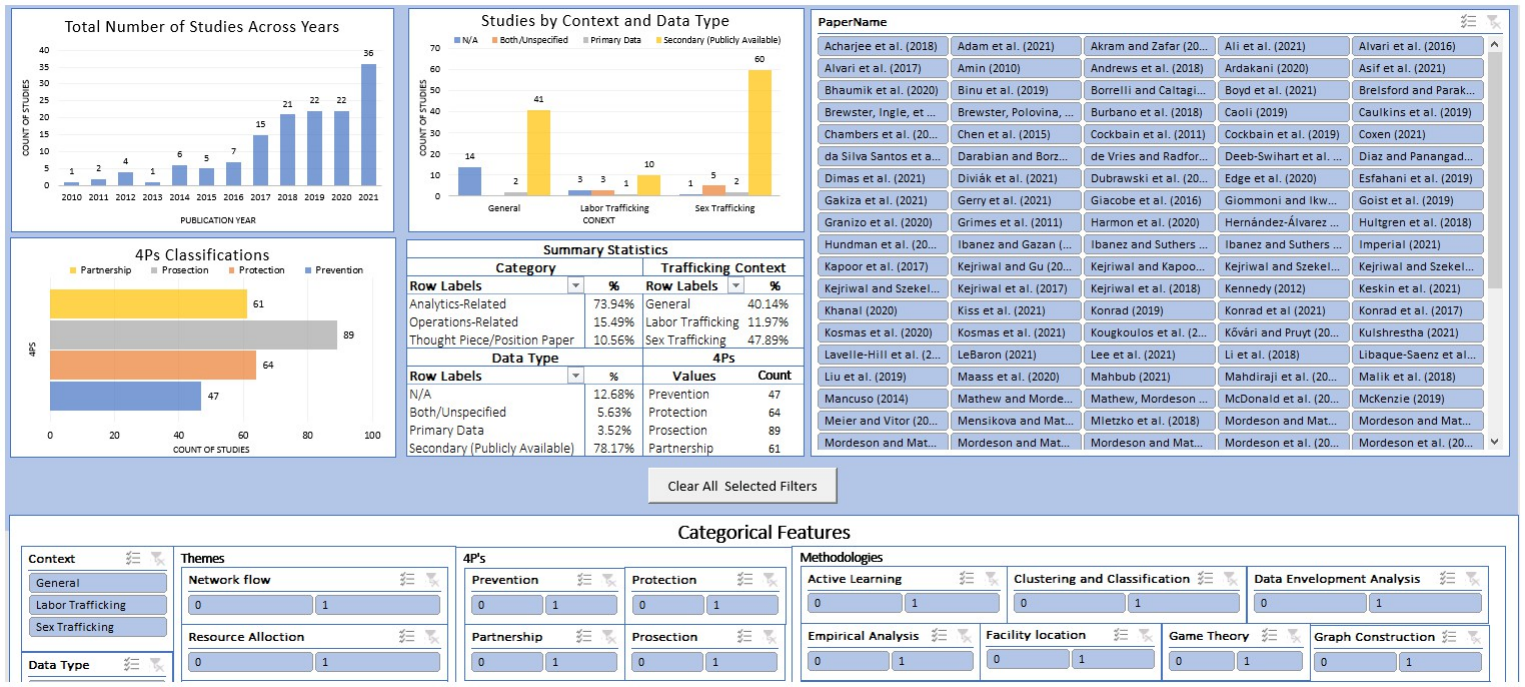
A screenshot of the spreadsheet tool created for closer examination of the set of 142 studies.

To compare studies across all three categories: Analytics, Operations Research, and Position / Thought Figs 7 and 8 are combined and the resulting figure can be found in the supplementary materials (see S1 Fig). We also provide a publicly available spreadsheet that can be used for closer examination of the individual studies, at https://github.com/gldimas/Dimas-et.-al-2022_Human-Trafficking-Literature-Review. This spreadsheet allows filtering studies on any combination of our nine features, returning all qualified studies. A screenshot of this tool can be see in Fig 9. As seen in this review, research at the intersection of anti-HT and OR and Applied Analytics is rapidly evolving and there is necessary complexity in the reviewing process. The selected keywords, search engines used and growing literature streams may impact the articles included in this review. In an effort to counter this limitation and support the longevity of this study, the authors have created an online submission form where individuals can submit works they believe to be related to the present study. Related submissions will be added to the dashboard tool on a semi-regular basis (see Fig 9) and will provide an evolving source of knowledge for researchers. The link for this form can be found on the github link provided earlier in this paragraph.

### Research question 3: What are the existing gaps and opportunities for future research?

Several gaps emerged based on the classification and grouping of all 142 studies in the set on the prime principles (4Ps) (Fig 6), methods (Figs 7 and 8), trafficking context (Fig 5), and resulting observations to Research questions 1 and 2. The following sections present opportunities for new avenues of investigation for OR and Analytics researchers in anti-HT efforts.

#### Broaden the typology and demographics of trafficking studied

The typology of trafficking activity, and by extension the demographic composition of victims, is diverse and under-explored in OR and Analytics [6]. As noted in [164], OR and Analytics researchers can increase the relevance and impact of their work by understanding the typology of trafficking and distinguish between various trafficking business models. To start, there is a clear need to expand the current focus to include labor trafficking. In OR and Analytics, sex trafficking (Fig 6) has received by far the most attention; while labor trafficking is estimated to account for over 60.0% of all trafficking instances [3], it constitutes only 12.0% of the reviewed studies. There is a close relationship between data source and type of HT studied. The absence of reliable, available data likely contributes to the lack of both analytically-based labor trafficking research, as well as the lack of diversity in data used in sex trafficking research; this is a well-documented issue across the anti-HT literature [22, 171–174]. The implications of the lack of data are also evident in studies using OR and Analytics methods and discussed further in the next section.

The diverse populations of those experiencing trafficking warrants further analysis. Nearly 7 7.0% of the studies observed were classified as applicable to Unspecified / All Individuals, indicating that the nuanced differences in victimology may be lacking. For example, no study we observed looked at trafficking through the lens of male victims or those who identify as LBGTQ+. These groups tend to be underrepresented in trafficking research despite their known presence [175, 176]. Inclusivity of more diverse victims and trafficker demographics in trafficking research expands insights into the unique characteristics, needs and behaviors across trafficking.

Beyond the demographics of those impacted by trafficking, it is apparent that there is an opportunity to expand the diversity of the demographics of trafficking locations. Whereas trafficking occurs globally in various facets of society, and differs in its appearance across cultures, many of the sex trafficking studies we reviewed were conducted within developed countries (approximately 78.0%). Thus, a clear opportunity exists to conduct anti-HT research in developing countries.

It is altogether possible that the lack of research identified in these areas may be a direct result of constrained factors such as the limited amount of data available. Even so, greater research diversity in trafficking typology and demographics will allow for an improved understanding of the extent and impact of trafficking worldwide, as well as greater insights into how future research can help address associated needs in the fight against HT. This brings us to the next research gap observed: the need to diversity data sources.

#### Diversify the data sources

Data collection and analysis have helped in the fight against HT in many ways, including the identification of HT victims [63, 134, 141], informing prevention campaigns [142], and detecting trafficking network behaviors [45, 80]. While thorough data analysis lays the necessary groundwork for such discoveries, it relies upon the utilization of a variety of data from disparate sources.

Over 78.0% of all studies in the set used secondary data sources exclusively (Fig 6). The use of secondary data is common across many domains and proves beneficial given that the data already exists, is oftentimes publicly available, and can provide researchers with large amounts of data they might not be able to obtain otherwise. In the context of the OR and Analytics studies observed, over 22.0% of the secondary data sources used were from the same source, the now defunct, backpage.com [170]. Of the secondary data sources, around 54.0% of these studies focused on sex trafficking. So, while secondary data sources have their place, as noted in the previous section the lack of data diversity compounds issues such as the typologies studied. Obtaining more robust data can broaden the information available, provide better insights into the scope of trafficking (such as prevalence), and examine changes that occur over time. Even with the limited current state of available data, OR and Analytics can still offer valuable contributions.

Researchers, particularly those in the Analytics community, could leverage collaborations and focus on developing the already existing, practitioner-collected data and help to operationalize it. For example, a common issue faced in anti-HT analysis pertains to missing and incomplete data [177]. Many analytical methods exist that could help bridge this gap and improve the current anti-HT data landscape. Additionally, although many disparate datasets are available and growing, the anti-HT community rarely leverages combined data sources in a way to assess the status, trends, and dynamics of trafficking activities, and is another avenue for future work.

Despite the usefulness of secondary data, they have drawbacks that may hinder the results of a study. Secondary data is often collected with another objective in mind and therefore the nuances of that data collection process may cause bias. For example, what constitutes trafficking may differ from study to study and is a well-documented issue across anti-HT research [178–180]. There may also be a context in which data simply does not exist. For these and other reasons, we advocate for the collection and use of primary data where possible. While primary data may be more resource-intensive to obtain, it provides researchers with curated data for their specific research goals. When possible, sharing this data within the anti-HT community provides additional resources for other work. In addition, embracing collaborations with practitioners or other researchers in the anti-HT domain throughout the data collection phase can serve to increase the quality of this data by providing domain-specific insights. Building such collaborations demonstrates effective employment of the partnership principle (the fourth P) and embodies our next recommendation.

#### Better inclusion and collaboration of the 4Ps

The 4Ps (prevention, protection, prosecution, and partnership) are widely acknowledged as a holistic set of principles that accounts for the spectrum of anti-HT efforts. To date, the majority of OR and Analytics studies in the set appear to be focused on prosecution (Fig 6). Thus, while there exists demonstrated impact for prosecution-related activities, there are opportunities to contribute to anti-HT efforts in the spheres of prevention, protection, and partnership. A key way to increase the impact of OR and Analytics research in the fight against HT is to be keenly aware of all stakeholders involved, their various objectives, and how the research addresses the 4Ps. For example, while law enforcement may make decisions based on the likelihood of prosecuting traffickers, possibly at the expense of additional trauma to victims, Non-Government Organizations (NGOs) may focus more on the immediate needs of the survivors, offering an avenue for research around prevention and protection.

NGOs and governmental agencies often work directly with victims and survivors and could both inform avenues for profitable research studies, and themselves benefit from collaboration with OR and Analytics researchers. Given the often extreme resource constraints under which NGOs and governmental organizations operate, examining ways to evaluate current operations and improve resource allocation is a direction that deserves more study; less than 3.0% of all studies considered these areas.

Beyond the scope of the present study, OR and Analytics can help in the fight against HT by looking at push factors associated with HT. These areas include and are not limited to poverty, abuse, and lack of resources to meet basic needs [181]. More broadly, looking at ways to help improve circumstances of vulnerable populations at risk of HT is a needed avenue for future research.

While there are many ways in which the OR and Analytics communities can apply methods in the fight against HT, researchers ought to judiciously evaluate the problem context at hand, and whether an off-the-shelf method is justified; likely, the context warrants in-depth understanding, so that proper methodologies can be developed to accurately model and address the trafficking context [164].

## Conclusions

This survey provides a synopsis of the current state of the literature in OR and Analytics approaches in anti-HT contexts by surveying the research methodologies adopted in studies published from 2010 through 2021. A total of 142 studies were included in the set and examined, demonstrating the ability and promise of applying analytical methods to advance the fight against HT. A number of themes arose after careful review of the features of these studies, thereby illustrating opportunities for future research. We observed an increasing trend in the number of studies for both OR and Analytics, thus demonstrating a growing awareness of the issue of HT. However, the tendency of these works to focus specifically on sex trafficking underscores the need for future research in labor trafficking. Very few (less than 2 4.0%) of the studies on anti-HT in OR and Analytics focus on a specific sub-population, potentially failing to consider the diverse needs of victims and survivors. Existing OR and Analytics studies echo the anti-HT community at large for more available data. HT is diverse and nuanced, and researchers should make careful considerations when adapting existing methods to this vexing societal issue, considering efforts equally in prevention, protection, prosecution, and partnership.

## Supporting information

S1 Checklist PRISMA 2020 checklist. (PDF)

S1 File Related studies. A list of related studies that fall outside of the scope of current work.
(PDF)

S1 Fig Granular view of theoretical and methodological topic inclusion for the set of 142 studies. The x-axis lists each study; and the y-axis depicts each of the Theoretical Approaches and Method. Operations Research studies appear on the far left (in blue), Analytics studies appear in the middle (in black) and Position / Thought studies appear on the far right (in red). If a study includes a given feature, the box is black, and grey otherwise. Theoretical approaches and methods are sorted in descending order based on the total count for each row.
(TIF)

## References

[1] Office to Monitor and Combat Trafficking in Persons, United States Department of State. 2019 trafficking in persons report; 2019. Available from: https://www.state.gov/reports/2019-trafficking-in-persons-report/.

[2] United Nations. Protocol to prevent, suppress and punish trafficking in persons; 2000. Available from: www.ohchr.org/en/professionalinterest/pages/protocoltraffickinginpersons.aspx.

[3] International Labour Organization. Global estimates of modern slavery: Forced labour and forced marriage; 2017. Available from: https://www.ilo.org/global/publications/books/WCMS_575479/lang-en/index.htm.

[4] International Labour Office. Profits and poverty: The economics of forced labour; 2014. Available from: https://www.ilo.org/global/topics/forced-labour/publications/profits-of-forced-labour-2014/lang-en/index.htm.

[5] Gallo M, Konrad RA, Thinyane H. An epidemiological perspective on labor trafficking. Journal of Human Trafficking. 2020; p. 1–22.32190715

[6] Polaris Project. The typology of modern slavery: Defining sex and labor trafficking in the United States; 2017. Available from: https://polarisproject.org/resources/the-typology-of-modern-slavery-defining-sex-and-labor-trafficking-in-the-united-states/.

[7] Amadasun S. Social work interventions for human trafficking victims’ in Nigeria. International Social Work. 2020;1:1–13.

[8] Okech D, Choi YJ, Elkins J, Burns AC. Seventeen years of human trafficking research in social work: A review of the literature. Journal of Evidence-Informed Social Work. 2018;15(2):103–122. doi: 10.1080/23761407.2017.1415177 29265959

[9] Potocky M. The travesty of human trafficking: A decade of failed US policy. Social Work. 2010;55(4):373–375. doi: 10.1093/sw/55.4.373 20977062

[10] Fraley HE, Aronowitz T, Stoklosa HM. Systematic review of human trafficking educational interventions for health care providers. Western Journal of Nursing Research. 2020;42(2):131–142. doi: 10.1177/0193945919837366 30924735

[11] Hemmings S, Jakobowitz S, Abas M, Bick D, Howard LM, Stanley N, et al. Responding to the health needs of survivors of human trafficking: A systematic review. BMC Health Services Research. 2016;16(1):1–9. doi: 10.1186/s12913-016-1538-8 27473258 PMC4966814

[12] McCarthy LA. Trafficking justice. Cornell University Press; 2016. Available from: https://www.cornellpress.cornell.edu/book/9781501701368/trafficking-justice/#bookTabs=1.

[13] Farrell A, Owens C, McDevitt J. New laws but few cases: Understanding the challenges to the investigation and prosecution of human trafficking cases. Crime, Law and Social Change. 2014;61(2):139–168. doi: 10.1007/s10611-013-9442-1

[14] Farrell A, Fahy S. The problem of human trafficking in the US: Public frames and policy responses. Journal of Criminal Justice. 2009;37(6):617–626. doi: 10.1016/j.jcrimjus.2009.09.010

[15] De Hert P, Muraszkiewicz J. Gary Becker and the economics of trafficking in human beings. New Journal of European Criminal Law. 2014;5(2):116–120. doi: 10.1177/203228441400500201

[16] Mahmoud TO, Trebesch C. The economics of human trafficking and labour migration: Micro-evidence from Eastern Europe. Journal of Comparative Economics. 2010;38(2):173–188. doi: 10.1016/j.jce.2010.02.001

[17] McAlpine A, Kiss L, Zimmerman C, Chalabi Z. Agent-based modeling for migration and modern slavery research: A systematic review. Journal of Computational Social Science. 2021;4(1):243–332. doi: 10.1007/s42001-020-00076-7

[18] Wen J, Klarin A, Goh E, Aston J. A systematic review of the sex trafficking-related literature: Lessons for tourism and hospitality research. Journal of Hospitality and Tourism Management. 2020;45:370–376. doi: 10.1016/j.jhtm.2020.06.001

[19] Mahalingam R. Human trafficking from a multidisciplinary perspectives: A literature review. Asian Journal of Social Science Research. 2019;2(2).

[20] Szablewska N, Kubacki K. Anti-human trafficking campaigns: A systematic literature review. Social Marketing Quarterly. 2018;24(2):104–122. doi: 10.1177/1524500418771611

[21] Schauer EJ, Wheaton EM. Sex trafficking into the United States: A literature review. Criminal Justice Review. 2006;31(2):146–169. doi: 10.1177/0734016806290136

[22] Konrad RA, Trapp AC, Palmbach TM, Blom JS. Overcoming human trafficking via operations research and analytics: Opportunities for methods, models, and applications. European Journal of Operational Research. 2017;259(2):733–745. doi: 10.1016/j.ejor.2016.10.049

[23] Kammer-Kerwick M, Busch-Armendariz N, Talley M. Disrupting illicit supply networks: New applications of operations research and data analytics to end modern slavery. Bureau of Business Research; 2018.

[24] Caulkins JP, Kammer-Kerwick M, Konrad R, Maass KL, Martin L, Sharkey T. A call to the engineering community to address human trafficking. National Academy of Engineering—The Bridge. 2019;49(3):68–73.

[25] Office to Monitor and Combat Trafficking in Persons. The 3Ps: Prevention, Protection, Prosecution; 2016. Online. Available from: https://2009-2017.state.gov/documents/organization/259311.pdf.

[26] Moher D, Liberati A, Tetzlaff J, Altman DG. Preferred Reporting Items for Systematic Reviews and Meta-Analyses: The PRISMA statement. PLoS Med. 2009;6(7):1–6. doi: 10.1371/journal.pmed.1000097 PMC2707599 19621072

[27] Raets S, Janssens J. Trafficking and technology: Exploring the role of digital communication technologies in the Belgian human trafficking business. European Journal on Criminal Policy and Research. 2021;27(2):215–238. doi: 10.1007/s10610-019-09429-z

[28] Farrell A, De Vries I. Measuring the nature and prevalence of human trafficking. The Palgrave International Handbook of Human Trafficking. 2020; p. 147–162. doi: 10.1007/978-3-319-63058-8_6

[29] Weitzer R. New directions in research on human trafficking. The ANNALS of the American Academy of Political and Social Science. 2014;653(1):6–24. doi: 10.1177/0002716214521562

[30] Acharjee S, Sarma DJ, Hanneman RA, Mordeson JN, Malik DS. Fuzzy soft attribute correlation coefficient and application to data of human trafficking. Proyecciones (Antofagasta). 2018;37(4):637–681. doi: 10.4067/S0716-09172018000400637

[31] Adam I, Al Qunaibit A, Shabeb N, Al Fehaid F, et al. Web Application based image geolocation analysis to detect human trafficking. Journal of Information Security and Cybercrimes Research. 2021;4(2):185–193.

[32] Akram M, Zafar F. A new approach to compute measures of connectivity in rough fuzzy network models. Journal of Intelligent & Fuzzy Systems. 2019;36(1):449–465. doi: 10.3233/JIFS-181751

[33] Ali S, Mathew S, Mordeson JN. Hamiltonian fuzzy graphs with application to human trafficking. Information Sciences. 2021;550:268–284. doi: 10.1016/j.ins.2020.10.029

[34] Alvari H, Shakarian P, Snyder JK. A non-parametric learning approach to identify online human trafficking. In: 2016 IEEE Conference on Intelligence and Security Informatics (ISI). IEEE; 2016. p. 133–138.

[35] Alvari H, Shakarian P, Snyder JK. Semi-supervised learning for detecting human trafficking. Security Informatics. 2017;6(1):1–14. doi: 10.1186/s13388-017-0029-8

[36] Andrews S, Brewster B, Day T. Organised crime and social media: A system for detecting, corroborating and visualising weak signals of organised crime online. Security Informatics. 2018;7(1):1–21. doi: 10.1186/s13388-018-0032-8

[37] Ardakani HM. Identifying human trafficking networks in Louisiana by using authorship attribution and network modeling. Louisiana State University; 2020. Available from: https://digitalcommons.lsu.edu/gradschool_dissertations/5274/.

[38] Asif M, Kattan DA, Pamučar D, Ali G. q-Rung orthopair fuzzy matroids with application to human trafficking. Discrete Dynamics in Nature and Society. 2021.

[39] Binu M, Mathew S, Mordeson JN. Connectivity index of a fuzzy graph and its application to human trafficking. Fuzzy Sets and Systems. 2019;360:117–136. doi: 10.1016/j.fss.2018.06.007

[40] Boyd DS, Perrat B, Li X, Jackson B, Landman T, Ling F, et al. Informing action for United Nations SDG target 8.7 and interdependent SDGs: Examining modern slavery from space. Humanities and Social Sciences Communications. 2021;8(1):1–14. doi: 10.1057/s41599-021-00792-z

[41] Burbano D, Hernández-Alvarez M. Illicit, hidden advertisements on Twitter. In: 2018 International Conference on eDemocracy & eGovernment (ICEDEG). IEEE; 2018. p. 317–321.

[42] Caoli Jr A. Machine learning in the analysis of social problems: The case of global human trafficking. The British University in Dubai; 2019. Available from: https://bspace.buid.ac.ae/handle/1234/1579.

[43] Chambers N, Forman T, Griswold C, Lu K, Khastgir Y, Steckler S. Character-based models for adversarial phone extraction: Preventing human sex trafficking. In: Proceedings of the 5th Workshop on Noisy User-Generated Text (W-NUT 2019); 2019. p. 48–56.

[44] Chen Q, De Arteaga M, Herlands W. Canonical autocorrelation analysis and graphical modeling for human trafficking characterization; 2015. Available from: https://www.cs.cmu.edu/~epxing/Class/10715/project-reports/ChenDeArteagaHerlands.pdf.

[45] Cockbain E, Brayley H, Laycock G. Exploring internal child sex trafficking networks using social network analysis. Policing: A Journal of Policy and Practice. 2011;5(2):144–157. doi: 10.1093/police/par025

[46] Cockbain E, Bowers K. Human trafficking for sex, labour and domestic servitude: How do key trafficking types compare and what are their predictors? Crime, Law and Social Change. 2019;72(1):9–34. doi: 10.1007/s10611-019-09836-7

[47] Coxen J. A Risk analysis and data driven approach to combating sex trafficking; University of Michigan; 2021. Available from: https://deepblue.lib.umich.edu/bitstream/handle/2027.42/170041/juliaoh_1.pdf.

[48] da Silva Santos M, Ladeira M, Van Erven GC, da Silva GL. Machine learning models to identify the risk of modern slavery in Brazilian cities. In: 2019 18th IEEE International Conference On Machine Learning And Applications (ICMLA). IEEE; 2019. p. 740–746.

[49] Darabian E, Borzooei RA. Results on vague graphs with applications in human trafficking. New Mathematics and Natural Computation. 2018;14(1):37–52. doi: 10.1142/S1793005718500047

[50] de Vries I, Radford J. Identifying online risk markers of hard-to-observe crimes through semi-inductive triangulation: The case of human trafficking in the United States. The British Journal of Criminology. 2022;62(3):639–658. doi: 10.1093/bjc/azab077

[51] Diaz M, Panangadan A. Natural language-based integration of online review datasets for identification of sex trafficking businesses. In: 2020 IEEE 21st International Conference on Information Reuse and Integration for Data Science (IRI). IEEE; 2020. p. 259–264. 10.1109/iri49571.2020.00044 PMC8631306 34853666

[52] Diviák T, Dijkstra JK, van der Wijk F, Oosting I, Wolters G. Women trafficking networks: Structure and stages of women trafficking in five Dutch small-scale networks. European Journal of Criminology. 2021; p. 14773708211053135.

[53] Dubrawski A, Miller K, Barnes M, Boecking B, Kennedy E. Leveraging publicly available data to discern patterns of human-trafficking activity. Journal of Human Trafficking. 2015;1(1):65–85. doi: 10.1080/23322705.2015.1015342

[54] Edge D, Yang W, Lytvynets K, Cook H, Galez-Davis C, Darnton H, et al. Design of a Privacy-Preserving Data Platform for Collaboration Against Human Trafficking. arXiv preprint arXiv:200505688. 2020.

[55] Esfahani SS, Cafarella MJ, Pouyan MB, DeAngelo G, Eneva E, Fano AE. Context-specific language modeling for human trafficking detection from online advertisements. In: Proceedings of the 57th Annual Meeting of the Association for Computational Linguistics; 2019. p. 1180–1184.

[56] Gakiza J, Jilin Z, Chang Kc, Tao L. Human trafficking solution by deep learning with keras and OpenCV. In: International Conference on Advanced Intelligent Systems and Informatics. Springer; 2021. p. 70–79.

[57] Giacobe NA, Altmire JB, Forster AE, Jackson AC, Raibick EW, Reep JA, et al. Characterizing sex trafficking in Pennsylvania for law enforcement. In: 2016 IEEE Symposium on Technologies for Homeland Security (HST). IEEE; 2016. p. 1–5.

[58] Giommoni L, Ikwu R. Identifying human trafficking indicators in the UK online sex market. Trends in Organized Crime. 2021; p. 1–24.

[59] Goist M, Chen THY, Boylan C. Reconstructing and analyzing the transnational human trafficking network. In: 2019 IEEE/ACM International Conference on Advances in Social Networks Analysis and Mining (ASONAM). IEEE; 2019. p. 493–500.

[60] Granizo SL, Caraguay ÁLV, López LIB, Hernández-Álvarez M. Detection of possible illicit messages using natural language processing and computer vision on Twitter and linked websites. IEEE Access. 2020;8:44534–44546. doi: 10.1109/ACCESS.2020.2976530

[61] Harmon R, Arnon D, Park B. TIP for tat: Political bias in human trafficking reporting. British Journal of Political Science. 2020; p. 1–11.

[62] Hernández-Álvarez M. Detection of possible human trafficking in Twitter. In: 2019 International Conference on Information Systems and Software Technologies (ICI2ST). IEEE; 2019. p. 187–191.

[63] Hultgren M, Whitney J, Jennex ME, Elkins A. A knowledge management approach to identify victims of human sex trafficking. Communications of the Association for Information Systems. 2018;42(1):23.

[64] Ibanez M, Gazan R. Detecting sex trafficking circuits in the US through analysis of online escort advertisements. In: 2016 IEEE/ACM International Conference on Advances in Social Networks Analysis and Mining (ASONAM). IEEE; 2016. p. 892–895.

[65] Ibanez M, Suthers DD. Detecting covert sex trafficking networks in virtual markets. In: 2016 IEEE/ACM International Conference on Advances in Social Networks Analysis and Mining (ASONAM). IEEE; 2016. p. 876–879.

[66] Ibanez M, Suthers DD. Detection of domestic human trafficking indicators and movement trends using content available on open internet sources. In: 2014 47th Hawaii International Conference on System Sciences. IEEE; 2014. p. 1556–1565.

[67] Imperial JM. How do pedophiles tweet? Investigating the writing styles and online personas of child cybersex traffickers in the Philippines. arXiv preprint arXiv:210709881. 2021.

[68] Kejriwal M, Ding J, Shao R, Kumar A, Szekely P. FlagIt: A system for minimally supervised human trafficking indicator mining. arXiv preprint arXiv:171203086. 2017.

[69] Kejriwal M, Szekely P. Information extraction in illicit web domains. In: Proceedings of the 26th International Conference on World Wide Web; 2017a. p. 997–1006.

[70] Kejriwal M, Szekely P. An investigative search engine for the human trafficking domain. In: International Semantic Web Conference. Springer; 2017b. p. 247–262.

[71] Kejriwal M, Szekely P. Knowledge graphs for social good: An entity-centric search engine for the human trafficking domain. IEEE Transactions on Big Data. 2017c.

[72] Kejriwal M, Szekely P, Knoblock C. Investigative knowledge discovery for combating illicit activities. IEEE Intelligent Systems. 2018;33(1):53–63. doi: 10.1109/MIS.2018.111144556

[73] Kejriwal M, Kapoor R. Network-theoretic information extraction quality assessment in the human trafficking domain. Applied Network Science. 2019;4(1):1–26. doi: 10.1007/s41109-019-0154-z

[74] Kejriwal M, Gu Y. Network-theoretic modeling of complex activity using UK online sex advertisements. Applied Network Science. 2020;5(1):1–23. doi: 10.1007/s41109-020-00275-1

[75] Kennedy E. Predictive patterns of sex trafficking online. Carnegie Mellon University; 2012. Available from: https://kilthub.cmu.edu/articles/thesis/Predictive_Patterns_of_Sex_Trafficking_Online/6686309/1.

[76] Kiss L, Fotheringhame D, Mak J, McAlpine A, Zimmerman C. The use of Bayesian networks for realist evaluation of complex interventions: Evidence for prevention of human trafficking. Journal of Computational Social Science. 2020;4(1):25–48. doi: 10.1007/s42001-020-00067-8

[77] Kulshrestha A. Detection of organized activity in online escort advertisements. McGill University (Canada); 2021. Available from: https://www.proquest.com/docview/2570357788?pq-origsite=gscholar&fromopenview=true.

[78] Lavelle-Hill R, Mazumder A, Goulding J, Smith G, Landman T. Machine learning methods for “Small-n, Large-p” problems: Understanding the complex drivers of modern-day slavery. Research Square preprint rs3rs-296275. 2021.

[79] Lee MC, Vajiac C, Kulshrestha A, Levy S, Park N, Jones C, et al. InfoShield: Generalizable information-theoretic human-trafficking detection. In: 2021 IEEE 37th International Conference on Data Engineering (ICDE). IEEE; 2021. p. 1116–1127.

[80] Li L, Simek O, Lai A, Daggett M, Dagli CK, Jones C. Detection and characterization of human trafficking networks using unsupervised scalable text template matching. In: 2018 IEEE International Conference on Big Data. IEEE; 2018. p. 3111–3120.

[81] Li R, Tobey M, Mayorga M, Caltagirone S, özaltın O. Detecting human trafficking: Automated classification of online customer reviews of massage businesses. Available at SSRN 3982796. 2021.

[82] Libaque-Saenz CF, Lazo J, Lopez-Yucra KG, Bravo ER. Could machine learning improve the prediction of child labor in Peru? In:Information Management and Big Data. Springer International Publishing; 2018. p. 15–30.

[83] Liu Y, Zhu L, Szekely P, Galstyan A, Koutra D. Coupled clustering of time-series and networks. In: Proceedings of the 2019 SIAM International Conference on Data Mining. SIAM; 2019. p. 531–539.

[84] Malik DS, Mathew S, Mordeson JN. Fuzzy incidence graphs: Applications to human trafficking. Information Sciences. 2018;447:244–255. doi: 10.1016/j.ins.2018.03.022

[85] Mancuso M. Not all madams have a central role: Analysis of a Nigerian sex trafficking network. Trends in Organized Crime. 2014;17(1):66–88. doi: 10.1007/s12117-013-9199-z

[86] Mathew S, Mordeson JN. Fuzzy influence graphs. New Mathematics and Natural Computation. 2017;13(3):311–325. doi: 10.1142/S1793005717400129

[87] Mathew S, Mordeson J, Yang HL. Incidence cuts and connectivity in fuzzy incidence graphs. Iranian Journal of Fuzzy Systems. 2019;16(2):31–43.

[88] McDonald GG, Costello C, Bone J, Cabral RB, Farabee V, Hochberg T, et al. Satellites can reveal global extent of forced labor in the world’s fishing fleet. Proceedings of the National Academy of Sciences. 2021;118(3). doi: 10.1073/pnas.2016238117 33431679 PMC7826370

[89] Mensikova A, Mattmann CA. Ensemble sentiment analysis to identify human trafficking in web data. In: Proceedings of ACM Workshop on Graph Techniques for Adversarial Activity Analytics (GTA32018); 2018. p. 1–5.

[90] Mletzko D, Summers L, Arnio AN. Spatial patterns of urban sex trafficking. Journal of Criminal Justice. 2018;58:87–96. doi: 10.1016/j.jcrimjus.2018.07.008

[91] Mordeson JN, Mathew S, Acharjee S. Aggregation operators: Applications to human trafficking and slavery. New Mathematics and Natural Computation. 2018;14(3):403–421. doi: 10.1142/S1793005718500242

[92] Mordeson JN, Mathew S, Binu M. Dialectic synthesis: Application to human trafficking. New Mathematics and Natural Computation. 2019;15(3):395–410. doi: 10.1142/S1793005719500224

[93] Mordeson JN, Mallenby M, Mathew S, Acharjee S. Human trafficking: Policy intervention. New Mathematics and Natural Computation. 2017;13(3):341–358. doi: 10.1142/S1793005717400142

[94] Mordeson JN, Mathew S. T-Norm fuzzy graphs. New Mathematics and Natural Computation. 2018;14(1):129–143. doi: 10.1142/S1793005718500096

[95] Mordeson JN, Mordeson JH, Mathew S. Directed Graphs Applied to Human Trafficking. New Mathematics and Natural Computation. 2018;14(3):445–455. doi: 10.1142/S1793005718500266

[96] Mordeson JN, Schwab-McCoy A, Mathew S, Binu M. Fuzzy indices with applications to human trafficking. New Mathematics and Natural Computation. 2019;15(3):411–425.

[97] Mordeson JN, Mathew S. Local look at human trafficking. New Mathematics and Natural Computation. 2017a;13(3):327–340. doi: 10.1142/S1793005717400130

[98] Mordeson JN, Mathew S. Non-deterministic flow in fuzzy networks and its application in identification of human trafficking chains. New Mathematics and Natural Computation. 2017b;13(3):231–243. doi: 10.1142/S1793005717400087

[99] Mordeson JN, Mathew S. Fuzzy logic applied to sustainable development goals and human trafficking. Symmetry. 2020a;12(1):87. doi: 10.3390/sym12010087

[100] Mordeson JN, Mathew S. Sustainable goals in combating human trafficking: Analysis by mathematics of uncertainty. Journal of Algebraic Hyperstructures and Logical Algebras. 2020b;1(1):49–59. doi: 10.29252/hatef.jahla.1.1.4

[101] Mukherjee S, Sederholm T, Roman AC, Sankar R, Caltagirone S, Ferres JL. A machine learning pipeline for aiding school identification from child trafficking images. arXiv preprint arXiv:210605215. 2021.

[102] Nagpal C, Miller K, Boecking B, Dubrawski A. An entity resolution approach to isolate instances of human trafficking online. arXiv preprint arXiv:150906659. 2015.

[103] Poelmans J, Elzinga P, Ignatov DI, Kuznetsov SO. Semi-automated knowledge discovery: Identifying and profiling human trafficking. International Journal of General Systems. 2012;41(8):774–804. doi: 10.1080/03081079.2012.721662

[104] Portnoff RS, Huang DY, Doerfler P, Afroz S, McCoy D. Backpage and bitcoin: Uncovering human traffickers. In: Proceedings of the 23rd ACM SIGKDD International Conference on Knowledge Discovery and Data Mining; 2017. p. 1595–1604.

[105] Prashad L, Dutta M, Dash BM. Spatial analysis of child labour in India. Journal of Children’s Services. 2021;16(4):269–280. doi: 10.1108/JCS-06-2019-0032

[106] Price CN. Constraining international human trafficking: A network analysis. Creighton University; 2016. Available from: https://www.proquest.com/docview/1839269136?pq-origsite=gscholar&fromopenview=true.

[107] Progga FT, Shahria MT, Arisha A, Shanto MUA. A deep learning based approach to child labour detection. In: 2020 6th Information Technology International Seminar (ITIS). IEEE; 2020. p. 24–29.

[108] Rabbany R, Bayani D, Dubrawski A. Active search of connections for case building and combating human trafficking. In: Proceedings of the 24th ACM SIGKDD International Conference on Knowledge Discovery & Data Mining; 2018. p. 2120–2129.

[109] Rodrigues DC, Prata DN, Silva MA. Exploring social data to understand child labor. International Journal of Social Science and Humanity. 2015;5(1):29. doi: 10.7763/IJSSH.2015.V5.416

[110] Sabon L, Yang S, Zhang Q. Social network analysis of a latino sex trafficking enterprise. Journal of Human Trafficking. 2021; p. 1–17. doi: 10.1080/23322705.2021.1898830

[111] Samanta S, Singhar SS, Gandomi AH, Ramasubbareddy S, Sankar S. A WiVi based IoT framework for detection of human trafficking victims kept in hideouts. In: International Conference on Internet of Things. Springer; 2020. p. 96–107.

[112] Sebastian A, Mordeson JN, Mathew S. Generalized fuzzy graph connectivity parameters with application to human trafficking. Mathematics. 2020;8(3):424. doi: 10.3390/math8030424

[113] Sethi RJ, Gil Y, Jo H, Philpot A. Large-scale multimedia content analysis using scientific workflows. In: Proceedings of the 21st ACM International Conference on Multimedia; 2013. p. 813–822.

[114] Silva DR, Philpot A, Sundararajan A, Bryan NM, Hovy E. Data integration from open internet sources and network detection to combat underage sex trafficking. In: Proceedings of the 15th Annual International Conference on Digital Government Research; 2014. p. 86–90.

[115] Simonson E. Semi-supervised classification of social media posts: Identifying sex-industry posts to enable better support for those experiencing sex-trafficking. arXiv preprint arXiv:210403233. 2021.

[116] Stylianou A, Schreier J, Souvenir R, Pless R. Traffickcam: Crowdsourced and computer vision based approaches to fighting sex trafficking. In: 2017 IEEE Applied Imagery Pattern Recognition Workshop (AIPR). IEEE; 2017. p. 1–8.

[117] Stylianou A, Xuan H, Shende M, Brandt J, Souvenir R, Pless R. Hotels-50k: A global hotel recognition dataset. In: Proceedings of the AAAI Conference on Artificial Intelligence. vol. 33; 2019. p. 726–733.

[118] Szakonyi A, Chellasamy H, Vassilakos A, Dawson M. Using technologies to uncover patterns in human trafficking. In: ITNG 2021 18th International Conference on Information Technology-New Generations. Springer; 2021. p. 497–502.

[119] Szekely P, Knoblock CA, Slepicka J, Philpot A, Singh A, Yin C, et al. Building and using a knowledge graph to combat human trafficking. In: International Semantic Web Conference. Springer; 2015. p.205–221.

[120] Tahir R, Imran MS, Minhas S, Sabahat N, Ilyas SHW, Gadi HR. Brick kiln detection and localization using deep learning techniques. In: 2021 International Conference on Artificial Intelligence (ICAI). IEEE; 2021. p. 37–43.

[121] Thöni A, Taudes A, Tjoa AM. An information system for assessing the likelihood of child labor in supplier locations leveraging Bayesian networks and text mining. Information Systems and e-Business Management. 2018;16(2):443–476. doi: 10.1007/s10257-018-0368-0

[122] Tong E, Zadeh A, Jones C, Morency LP. Combating human trafficking with multimodal deep models. In: Proceedings of the 55th Annual Meeting of the Association for Computational Linguistics (Volume 1: Long Papers). Vancouver, Canada: Association for Computational Linguistics; 2017. p. 1547–1556.

[123] Upadhayay B, Lodhia ZAM, Behzadan V. Combating human trafficking via automatic OSINT collection, Validation and Fusion. In: Workshop Proceedings of the 15th International AAAI Conference on Web and Social Media; 2020.

[124] Vogt AA. Combating human trafficking using mathematics. In: Undergraduate Research and Scholarship Symposium. Duquesne University; 2016. Available from: https://dsc.duq.edu/urss/2016/proceedings/3.

[125] Wang L, Laber E, Saanchi Y, Caltagirone S. Sex trafficking detection with ordinal regression neural networks. arXiv preprint arXiv:190805434. 2019.

[126] Wang H, Cai C, Philpot A, Latonero M, Hovy EH, Metzler D. Data integration from open internet sources to combat sex trafficking of minors. In: Proceedings of the 13th Annual International Conference on Digital Government Research; 2012. p. 246–252.

[127] Whitney JC. KM vs human trafficking: An exploratory study on using emojis for a knowledge driven approach to identifying online human sex trafficking. San Diego State University; 2017. Available from: https://digitallibrary.sdsu.edu/islandora/object/sdsu%3A21609.

[128] White A, Guikema S, Carr B. Why are you here? Modeling illicit massage business location characteristics with machine learning. Journal of Human Trafficking. 2021; p. 1–21. doi: 10.1080/23322705.2021.1982238

[129] Wiriyakun C, Kurutach W. Extracting co-occurrences of emojis and words as important features for human trafficking detection models. Journal of Intelligent Informatics and Smart Technology. 2021a;7.

[130] Wiriyakun C, Kurutach W. Feature selection for human trafficking detection models. In: 2021 IEEE/ACIS 20th International Fall Conference on Computer and Information Science (ICIS Fall). IEEE; 2021b. p. 131–135.

[131] Yang Y, Hu X, Liu H, Zhang J, Li Z, Yu PS. Understanding and monitoring human trafficking via social sensors: A sociological approach. arXiv preprint arXiv:180510617. 2018.

[132] Yao Y, Liu Y, Guan Q, Hong Y, Wang R, Wang R, et al. Spatiotemporal distribution of human trafficking in China and predicting the locations of missing persons. Computers, Environment and Urban Systems. 2021;85:101567. doi: 10.1016/j.compenvurbsys.2020.101567

[133] Zhou AJ, Luo J, McGibbney LJ. Multimedia metadata-based forensics in human trafficking web data. In: 2016 Workshop on Search and Exploration of X-rated Information. WSDM; 2016. p. 10–14.

[134] Zhu J, Li L, Jones C. Identification and detection of human trafficking using language models. In: 2019 European Intelligence and Security Informatics Conference (EISIC). IEEE; 2019. p. 24–31.

[135] Bhaumik A, Roy SK, Weber GW. Hesitant interval-valued intuitionistic fuzzy-linguistic term set approach in Prisoners’ dilemma game theory using TOPSIS: A case study on human-trafficking. Central European Journal of Operations Research. 2020;28(2):797–816. doi: 10.1007/s10100-019-00638-9

[136] Brelsford J, Parakh S. A systems modeling approach to analyzing human trafficking. In: 2018 Winter Simulation Conference (WSC). IEEE; 2018. p. 12–21.

[137] Dimas GL, Khalkhali ME, Bender A, Maass KL, Konrad R, Blom JS, et al. Estimating effectiveness of identifying human trafficking via data envelopment analysis. arXiv preprint arXiv:201207746. 2021.

[138] F Gerry QC, Vallabhaneni S, Shaw P. Game theory and the human trafficking dilemma. Journal of Human Trafficking. 2021;7(2):168–186. doi: 10.1080/23322705.2019.1688086

[139] Grimes J, Dillon RL, Tinsley CH. Systems dynamics as a method for analyzing human trafficking. In: 2011 Systems Dynamics Society; 2011. Available from: https://proceedings.systemdynamics.org/2011/proceed/papers/P1131.pdf.

[140] Kapoor R, Kejriwal M, Szekely P. Using contexts and constraints for improved geotagging of human trafficking webpages. In: Proceedings of the Fourth International ACM Workshop on Managing and Mining Enriched Geo-Spatial Data; 2017. p. 1–6.

[141] Keskin BB, Bott GJ, Freeman NK. Cracking sex trafficking: Data analysis, pattern recognition, and path prediction. Production and Operations Management. 2021;30(4):1110–1135. doi: 10.1111/poms.13294

[142] Konrad RA. Designing awareness campaigns to counter human trafficking: An analytic approach. Socio-Economic Planning Sciences. 2019;67:86–93. doi: 10.1016/j.seps.2018.10.005

[143] Kosmas D, Sharkey TC, Mitchell JE, Maass KL, Martin L. Interdicting restructuring networks with applications in illicit trafficking. arXiv preprint arXiv:201107093. 2020.

[144] Kosmas D, Melander C, Singerhouse E, Sharkey TC, Maass KL, Barrick K, et al. Generating synthetic but realistic human trafficking networks for modeling disruptions through transdisciplinary and community-based action research. arXiv preprint arXiv:220301893. 2022.

[145] Kougkoulos I, Cakir MS, Kunz N, Boyd DS, Trautrims A, Hatzinikolaou K, et al. A multi-method approach to prioritize locations of labor exploitation for ground-based interventions. Production and Operations Management. 2021.

[146] Kővári A, Pruyt E. A model-based exploration and policy analysis related to prostitution and human trafficking. International Journal of System Dynamics Applications (IJSDA). 2014;3(4):36–64. doi: 10.4018/ijsda.2014100103

[147] Maass KL, Trapp AC, Konrad R. Optimizing placement of residential shelters for human trafficking survivors. Socio-Economic Planning Sciences. 2020;70:100730. doi: 10.1016/j.seps.2019.100730

[148] Mahdiraji HA, Hafeez K, Jafarnejad A, Rezayar A. An analysis of the impact of negative CSR ‘forced labour’ parameter on the profitability of supply chain contracts. Journal of Cleaner Production. 2020;271:122274. doi: 10.1016/j.jclepro.2020.122274

[149] Mahbub N. An approach to effective disruption of human trafficking through data-driven spatial-temporal prevalence estimation and optimal interdiction of interdependent illicit trades. State University of New York at Buffalo; 2021. Available from: https://www.proquest.com/docview/2582070644?pq-origsite=gscholar&fromopenview=true.

[150] Meier S, Vitor F. Developing a discrete event simulation model to overcome human trafficking. In: IIE Annual Conference. Proceedings. Institute of Industrial and Systems Engineers (IISE); 2021. p. 7–12.

[151] Neely B, Senft E, Turner B, Weeks B, Palmer J, Caddell J. Leveraging system dynamics and value modeling to identify strategic priorities to combat human trafficking in India. In: Proceedings of the Annual General Donald R. Keith Memorial Conference. Society for Industrial and Systems Engineering; 2019. p. 19–37.

[152] Ramchandani P, Bastani H, Wyatt E. Unmasking human trafficking risk in commercial sex supply chains with machine learning. Available at SSRN 3866259. 2021.

[153] Senft E, Weeks B, Palmer J, Neely B, Turner B, Caddell J. A systems dynamics approach to human trafficking in Maharashtra,India. In: 2019 IEEE International Systems Conference (SysCon). IEEE; 2019. p. 1–7.

[154] Stapleton A, Chisholm N, Stapleton L. Sex trafficking from a supply chain systems perspective. IFAC Proceedings Volumes. 2012;45(10):15–20. doi: 10.3182/20120611-3-IE-4029.00006

[155] Taylor KC. Teaching decision-making and building resilience in youth–A case study to reduce the supply of vulnerable youth to sex traffickers in Atlanta, Georgia. European Journal of Operational Research. 2018;268(3):960–970. doi: 10.1016/j.ejor.2017.11.067

[156] Tezcan B, Maass KL. Human trafficking interdiction with decision dependent success. engrXiv preprint engrXiv:dt8fs. 2020.

[157] Amin S. A step towards modeling and destabilizing human trafficking networks using machine learning methods. In: 2010 AAAI Spring Symposium Series; 2010.

[158] Borrelli D, Caltagirone S. Non-traditional cyber adversaries: Combatting human trafficking through data science. Cyber Security. 2020;4(1):77–90.

[159] Brewster B, Ingle T, Rankin G. Crawling open-source data for indicators of human trafficking. In: 2014 IEEE/ACM 7th International Conference on Utility and Cloud Computing. IEEE; 2014. p. 714–719.

[160] Brewster B, Polovina S, Rankin G, Andrews S. Knowledge management and human trafficking: Using conceptual knowledge representation, text analytics and open-source data to combat organized crime. In: International Conference on Conceptual Structures. Springer; 2014. p. 104–117.

[161] Deeb-Swihart J, Endert A, Bruckman A. Understanding law enforcement strategies and needs for combating human trafficking. In: Proceedings of the 2019 CHI Conference on Human Factors in Computing Systems; 2019. p. 1–14.

[162] Hundman K, Gowda T, Kejriwal M, Boecking B. Always lurking: Understanding and mitigating bias in online human trafficking detection. In: Proceedings of the 2018 AAAI/ACM Conference on AI, Ethics, and Society; 2018. p. 137–143.

[163] Khanal S. Human trafficking in Nepal: Can big data help? Undergraduate Research Journal;24(1):5.

[164] Konrad RA, Trapp AC, Maass KL, Dimas GL. Perspectives on how to conduct responsible anti-human trafficking research in operations and analytics. arXiv preprint arXiv:200616445. 2021.

[165] LeBaron G. The role of supply chains in the global business of forced labour. Journal of Supply Chain Management. 2021;57(2):29–42. doi: 10.1111/jscm.12258

[166] McKenzie J. Identifying and mitigating human trafficking risk through the use of financial institutions. Utica College; 2019. Available from: https://www.proquest.com/docview/2228305608?pq-origsite=gscholar&fromopenview=true.

[167] Orantes M. Leveraging machine learning and artificial intelligence to combat human trafficking. Utica College; 2018. Available from: https://www.proquest.com/docview/2160987666?pq-origsite=gscholar&fromopenview=true.

[168] Tambe P, Tambay P. Reducing modern slavery using AI and blockchain. In: 2020 IEEE/ITU International Conference on Artificial Intelligence for Good (AI4G). IEEE; 2020. p. 22–27.

[169] Weinberg N, Bora A, Sassetti F, Bryant K, Rootalu E, Bikziantieieva K, et al. AI against modern slavery: Digital insights into modern slavery reporting—Challenges and opportunities. In: AI for Social Good, Association for the Advancement of Artificial Intelligence Fall Symposium 2020. AAAI; 2020.

[170] United States Justice Department. Justice Department leads effort to seize Backpage.com, the internet’s leading forum for prostitution ads, and obtains 93-count federal indictment; 2018. Available from: https://www.justice.gov/opa/pr/justice-department-leads-effort-seize-backpagecom-internet-s-leading-forum-prostitution-ads.

[171] Sweileh WM. Research trends on human trafficking: A bibliometric analysis using Scopus database. Globalization and Health. 2018;14(1):1–12. doi: 10.1186/s12992-018-0427-9 30409223 PMC6225706

[172] Farrell A, Reichert J. Using US law-enforcement data: Promise and limits in measuring human trafficking. Journal of Human Trafficking. 2017;3(1):39–60. doi: 10.1080/23322705.2017.1280324

[173] Gozdiak EM. Data and research on human trafficking: Bibliography of research-based literature. Washington, DC: Institute for the Study of International; 2011.

[174] Laczko F. Human trafficking: The need for better data. Migration Information Source. 2002;1:61–80.

[175] Quintana NS, Rosenthal J, Krehely J. On the streets: The federal response to gay and transgender homeless youth. Washington, DC: Center for American Progress; 2010.

[176] Freeman L, Hamilton D. A count of homeless youth in New York City. New York: Empire State Coalition of Youth and Family Services; 2008.

[177] Ali HM. Data collection on victims of human trafficking: An analysis of various sources. Journal of Human Security. 2010;6(1):55–69. doi: 10.3316/JHS0601055

[178] Aghazarm C, Laczko F. Human trafficking: New directions for research. Geneva International Organization for Migration; 2008.

[179] Alvarez MB, Alessi EJ. Human trafficking is more than sex trafficking and prostitution: Implications for social work. Affilia. 2012;27(2):142–152. doi: 10.1177/0886109912443763

[180] Gibbons P, Chisolm-Straker M, Stoklosa H. Human trafficking: Definitions, epidemiology, and shifting ground. Medical Perspectives on Human Trafficking in Adolescents. 2020; p. 1–12. doi: 10.1007/978-3-030-43367-3_1

[181] Kerr P. Push and pull: The intersections of poverty, health disparities and human trafficking. Public Health & Social Justice. 2014;3(2):1–5.

