# Supplementary material for: Operations research and analytics to combat human trafficking: A systematic review of academic literature
Source: PLoS One. 2022 Aug 29;17(8):e0273708. doi: 10.1371/journal.pone.0273708 (PMC9423650; doi:10.1371/journal.pone.0273708)
Supplement: S1 File — A list of related studies that fall outside of the scope of current work. (PDF) [file pone.0273708.s002.pdf]

Supplementary Materials 2: Related Studies

References

1. Bedford DA, Bekbalaeva J, Ballard KM, Hernandez TJ. Global human trafficking seen through the lens of semantics and text analytics. Proceedings of the Association for Information Science and Technology. 2017;54(1):535–538.

2. Bliss N, Briers M, Eckstein A, Goulding J, Lopresti DP, Mazumder A, et al. CCC/Code 8.7: Applying AI in the fight against modern slavery. arXiv preprint arXiv:210613186. 2021;.

3. Chandaliya PK, Garg P, Nain N. Retrieval of facial images re-rendered with natural aging effect using child facial image and age. In: 2018 14th International Conference on Signal-Image Technology & Internet-Based Systems (SITIS). IEEE; 2018. p. 457–463.

4. Chandaliya PK, Nain N. Child face age progression and regression using self-attention multi-scale patch GAN. In: 2021 International Joint Conference on Biometrics (IJCB); 2021. p. 1–8.

5. Chan L, Silverman BW, Vincent K. Multiple systems estimation for sparse capture data: Inferential challenges when there are non-overlapping lists. Journal of the American Statistical Association. 2021;116(535):1297–1306.

6. Cho SH, Fang X, Tayur S, Xu Y. Combating child labor: Incentives and information disclosure in global supply chains. Manufacturing & Service Operations Management. 2019;21(3):692–711.

7. Christ KL, Helliar CV. Blockchain technology and modern slavery: Reducing deceptive recruitment in migrant worker populations. Journal of Business Research. 2021;131:112–120.

8. Chung W, Mustaine E, Zeng D. Criminal intelligence surveillance and monitoring on social media: cases of cyber-trafficking. In: 2017 IEEE International Conference on Intelligence and Security Informatics (ISI). IEEE; 2017. p. 191–193.

9. Cockbain E, Bowers K. Human trafficking for sex, labour and domestic servitude: How do key trafficking types compare and what are their predictors? Crime, Law and Social Change. 2019;72(1):9–34.

10. Coxen J, Castro V, Carr B, Bredin G, Guikema S. COVID-19 pandemic's impact on online sex advertising and sex trafficking; 2021. Available from: `https://osf.io/preprints/socarxiv/tfyj5/`.

11. de Azevedo Drummond P. Optimization of port-of-entry operation in the US: An anti-human trafficking focus; 2021. Available from: `https://www.proquest.com/docview/2595490826?pq-origsite=gscholar&fromopenview=true`.

12. Deb D, Aggarwal D, Jain AK. Identifying missing children: Face age-progression via deep feature aging. In: 2020 25th International Conference on Pattern Recognition (ICPR). IEEE; 2021. p. 10540–10547.

13. Diviák T, Dijkstra JK, van der Wijk F, Oosting I, Wolters G. Women trafficking networks: Structure and stages of women trafficking in five Dutch small-scale networks. European Journal of Criminology. 2021; p. 14773708211053135.

14. Dubrawski A. Machine Learning for adaptable heterogeneous indexing and search. Carnegie Mellon University Pittsburgh United States; 2018. Available from: https://apps.dtic.mil/sti/pdfs/AD1060877.pdf.

15. Elliott S, Denise Smith M. Simulating a multi-agency approach for the protection of trafficked persons in migration and displacement settings. Journal of Human Trafficking. 2020;6(2):168–181.

16. Farrell A, De Vries I. Measuring the nature and prevalence of human trafficking. The Palgrave International Handbook of Human Trafficking. 2020; p. 147–162.

17. Forman T, Chambers N. Extracting phone numbers from adversarial & visually corrupted text;. Available from: https://vigilworkshop.github.io/static/papers-2021/15.pdf.

18. Grimes J, Dillon RL, Tinsley CH. System Dynamics as a method for analyzing human trafficking. Systems Dynamics Society. 2011;.

19. Hammack AR. Does neighborhood matter? Examining geographical correlates of human trafficking influenced areas in Colorado Springs, 2014-2019; 2021. Available from: https://www.proquest.com/docview/2620405497?pq-origsite=gscholar&fromopenview=true.

20. Hernández-Álvarez M, Granizo SL. Detection of human trafficking ads in Twitter using natural language processing and image processing. In: International Conference on Applied Human Factors and Ergonomics. Springer; 2020. p. 77–83.

21. Hewener H, Risser C, Brausch L, Rohrer T, Tretbar S. A mobile ultrasound system for majority detection. In: 2019 IEEE International Ultrasonics Symposium (IUS). IEEE; 2019. p. 502–505.

22. Hultgren MR. An exploratory study of the indicators of trafficking in online female escort ads; 2015. Available from: https://digitallibrary.sdsu.edu/islandora/object/sdsu%3A1940.

23. Hultgren M, Jennex ME, Persano J, Ornatowski C. Using knowledge management to assist in identifying human sex trafficking. In: 2016 49th Hawaii International Conference on System Sciences (HICSS). IEEE; 2016. p. 4344–4353.

24. Ibanez M, Suthers DD. Detection of domestic human trafficking indicators and movement trends using content available on open internet sources. In: 2014 47th Hawaii International Conference on System Sciences (HICSS). IEEE; 2014. p. 1556–1565.

25. Ibanez M, Gazan R. Virtual indicators of sex trafficking to identify potential victims in online advertisements. In: 2016 IEEE/ACM International Conference on Advances in Social Networks Analysis and Mining (ASONAM). IEEE; 2016. p. 818–824.

26. Kejriwal M, Szekely P. Technology-assisted investigative search: A case study from an illicit domain. In: Extended Abstracts of the 2018 CHI Conference on Human Factors in Computing Systems; 2018. p. 1–9.

27. Kidd RM. Vulnerability, victimization and VIVA: A cluster analysis of cross-national human trafficking victims. Virginia Tech; 2021. Available from: https://vtechworks.lib.vt.edu/handle/10919/103246.

28. Konrad R, Trapp A, Maass KL. Human trafficking analysis. OR/MS Today. 2017;44(2):30–33.

29. Kostakos P, Špráchalová L, Pandya A, Aboeleinen M, Oussalah M. Covert online ethnography and machine learning for detecting individuals at risk of being drawn into online sex work. In: 2018 IEEE/ACM International Conference on Advances in Social Networks Analysis and Mining (ASONAM). IEEE; 2018. p. 1096–1099.

30. Saenz CFL, Lazo JGL, López-Yucra KG, Bravo E. Predicting Child Labor in Peru: A comparison of Logistic Regression and Neural Networks Techniques. In: SIMBig; 2017.Available from: https://www.semanticscholar.org/paper/Predicting-Child-Labor-in-Peru%3A-A-comparison-of-and-Saenz-Lazo/9f468361346dab87fd4977f4f764da69931267bd.

31. L'Hoiry X, Moretti A, Antonopoulos GA. Identifying sex trafficking in adult services websites: An exploratory study with a British police force. Trends in Organized Crime. 2021; p. 1–22.

32. Liu CYJ. Facial identification from online images for use in the prevention of child trafficking and exploitation. Liverpool John Moores University (United Kingdom); 2018. Available from: https://www.proquest.com/docview/2495344687?pq-origsite=gscholar&fromopenview=true.

33. Lopez JJ, Truesdale-Moore S. The Use of spatial statistics to control human and sex trafficking. International Journal of Social Science and Humanity. 2020;10(2).

34. Mantell S, Kaya YB, Maass KL, Konrad R, Trapp AC, Dimas GL, et al. Discrete event simulation to evaluate shelter capacity expansion options for LGBTQ+ homeless youth. arXiv preprint arXiv:220413162. 2022;.

35. Mayorga M, Tateosian L, Velasquez G, Amindarbari R, Caltagirone S. Countering human trafficking using ISE/OR techniques. In: Emerging Frontiers in Industrial and Systems Engineering. CRC Press; 2019. p. 237–257.

36. McAlpine A, Kiss L, Zimmerman C, Chalabi Z. Agent-based modeling for migration and modern slavery research: A systematic review. Journal of Computational Social Science. 2021;4(1):243–332.

37. Mletzko D, Summers L, Arnio AN. Spatial patterns of urban sex trafficking. Journal of Criminal Justice. 2018;58:87–96.

38. Nagurney A. Operations research perspectives. Operations Research. 2022;9:100233.

39. Nath R, Das M. Women trafficking problem in Assam: The behavioral game aspects. International Journal of Applied Behavioral Economics (IJABE). 2021;10(3):12–40.

40. Orantes M. Leveraging machine learning and artificial intelligence to combat human trafficking; 2018. Available from: https://www.proquest.com/docview/2160987666?pq-origsite=gscholar&fromopenview=true.

41. Prashad L, Dutta M, Dash BM. Spatial analysis of child labour in India. Journal of Children's Services. 2021;16(4):269–280. doi:https://doi.org/10.1108/JCS-06-2019-0032.

42. Raets S, Janssens J. Trafficking and technology: Exploring the role of digital communication technologies in the Belgian human trafficking business. European Journal on Criminal Policy and Research. 2021;27(2):215–238.

43. Rodríguez JI, Durán SR, Díaz-López D, Pastor-Galindo J, Mármol FG. C 3-Sex: A conversational agent to detect online sex offenders. Electronics. 2020;9(11):1779.

44. Sabo T, Pilz A. Using SAS® text analytics to assess international human trafficking patterns. In: Proceedings of the SAS Global Forum 2018 Conference.; 2018.Available from: https://www.sas.com/content/dam/SAS/support/en/sas-global-forumproceedings/2018/1986-2018.pdf.

45. Sharkey TC, Maass K, Song Y, Barrick K, Farrell A, Martin L. Better together: A transdisciplinary approach to disrupt human trafficking. Industrial Engineer. 2021;53(11).

46. Shishira SS, Patil MJS. Detection of illicit Messages in Twitter using support vector machine and VGG16. Information Technology in Industry. 2021;9(3):794–804.

47. Stockhem O. Improving the international regulation of cybersex trafficking of women and children through the use of data science and artificial intelligence. Global Campus of Human Rights; 2020. Available from: https://repository.gchumanrights.org/server/api/core/bitstreams/c97da93c-5933-4bcc-8f6d-4e5282f0efa0/content.

48. Stylianou A. Learning about large scale image search: Lessons from global scale hotel recognition to fight sex trafficking. Washington University in St. Louis; 2018. Available from: https://www.proquest.com/docview/2155413766?pq-origsite=gscholar&fromopenview=true.

49. Stylianou A, Norling-Ruggles A, Souvenir R, Pless R. Indexing open imagery to create tools to fight sex trafficking. In: 2015 IEEE Applied Imagery Pattern Recognition Workshop (AIPR). IEEE; 2015. p. 1–6.

50. Stylianou A, Souvenir R, Pless R. TraffickCam: Explainable image matching for sex trafficking investigations. arXiv preprint arXiv:191003455. 2019;.

51. Thöni A. Sustainability risk monitoring in supply chains: Ranking suppliers using text mining and Bayesian networks with a focus on child labor; 2015. Available from: https://repositum.tuwien.at/handle/20.500.12708/9047.

52. Tueller SJ, Gibbs DA, Kluckman MN. Estimating unidentified sex trafficking in the child welfare population. Journal of Human Trafficking. 2021; p. 1–13.

53. Unertl KM, Walsh CG, Clayton EW. Combatting human trafficking in the United States: How can medical informatics help? Journal of the American Medical Informatics Association. 2021;28(2):384–388.

54. Van Buren III HJ, Schrempf-Stirling J, Westermann-Behaylo M. Business and human trafficking: A social connection and political responsibility model. Business & Society. 2021;60(2):341–375.

55. Vincent K, Zhang SX, Dank M. Searching for sex trafficking victims: Using a novel link-tracing method among commercial sex workers in Muzaffarpur, India. Crime & Delinquency. 2021;67(13-14):2254–2277.

56. Volodko A, Cockbain E, Kleinberg B. “Spotting the signs” of trafficking recruitment online: Exploring the characteristics of advertisements targeted at migrant job-seekers. Trends in Organized Crime. 2020;23(1):7–35.

57. Wang H, Cai C, Philpot A, Latonero M, Hovy EH, Metzler D. Data integration from open internet sources to combat sex trafficking of minors. In: Proceedings of the 13th Annual International Conference on Digital Government Research; 2012. p. 246–252.

58. Wang H, Philpot A, Hovy E, Latonero M. Data Mining and integration to combat child trafficking. Retrieved from Carnegie Mellon University, School of Computer Science website: http://www cs cmu edu/˜ hovy/papers/12dgo-trafficking pdf. 2014;.

59. Weinberg N, Bora A, Sassetti F, Bryant K, Rootalu E, Bikziantieieva K, et al.. AI against modern slavery: Digital insights into modern slavery reporting-challenges and opportunities; 2020. Available from: https://fra1.digitaloceanspaces.com/wikirateproject/files/7398/22246.pdf.

60. Weitzer R. New directions in research on human trafficking. The ANNALS of the American Academy of Political and Social Science. 2014;653(1):6–24.

61. Whitehead J, Jackson J, Balch A, Francis B. On the unreliability of multiple systems estimation for estimating the number of potential victims of modern slavery in the UK. Journal of Human Trafficking. 2021;7(1):1–13.

62. Xian LY, Logeswaran R. Human trafficking through data sharing and analytics. In: 2022 IEEE International Conference on Distributed Computing and Electrical Circuits and Electronics (ICDCECE). IEEE; 2022. p. 1–4.

63. Xie X, Aros-Vera F. An interdependent network interdiction model for disrupting sex trafficking networks. Production and Operations Management. 2022;31(6):2695–2713.
