# Supplementary figures and images for: Operations research and analytics to combat human trafficking: A systematic review of academic literature
Source: PLoS One. 2022 Aug 29;17(8):e0273708. doi: 10.1371/journal.pone.0273708 (PMC9423650; doi:10.1371/journal.pone.0273708)

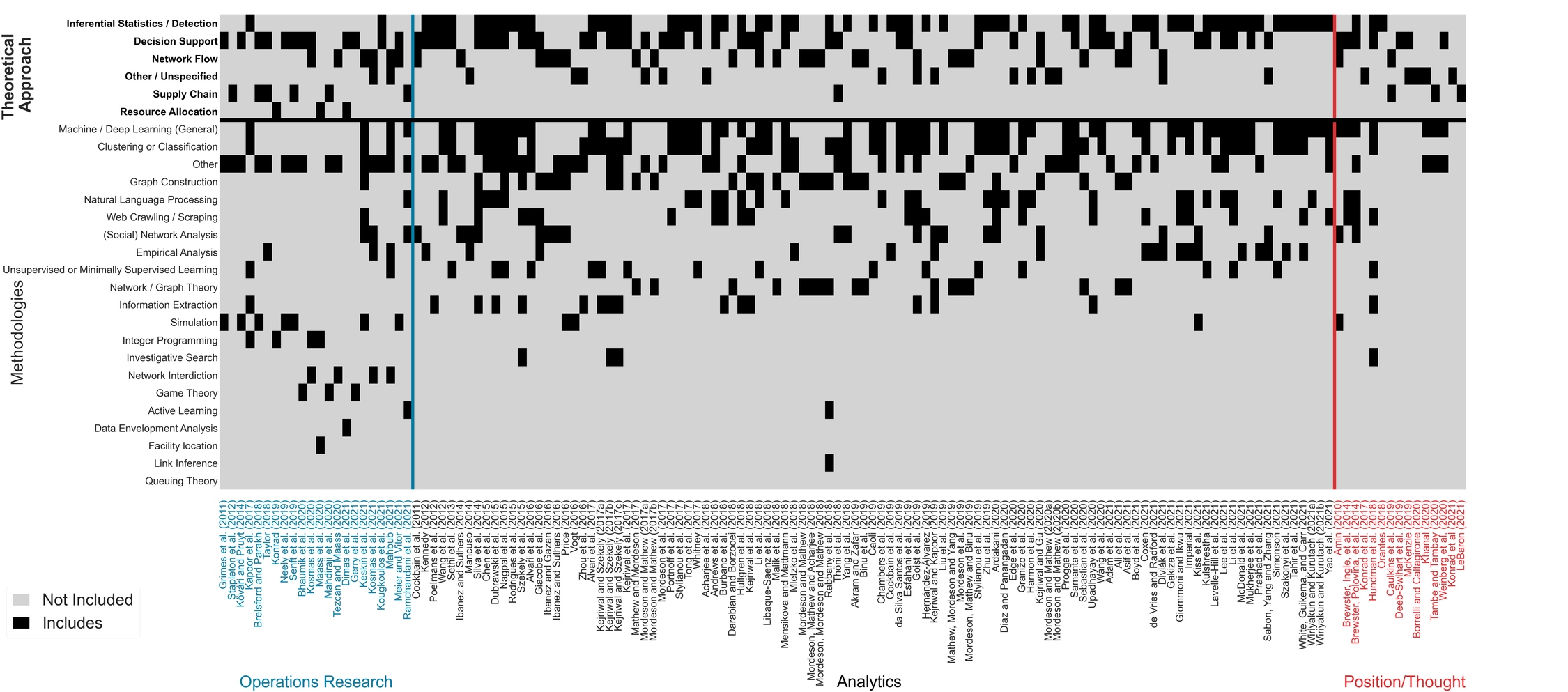

Supplement: S1 Fig — The x-axis lists each study; and the y-axis depicts each of the Theoretical Approaches and Method. Operations Research studies appear on the far left (in blue), Analytics studies appear in the middle (in black) and Position / Thought studies appear on the far right (in red). If a study includes a given feature, the box is black, and grey otherwise. Theoretical approaches and methods are sorted in descending order based on the total count for each row. (TIF) [file pone.0273708.s003.tif]
